# Single-cell analyses reveal novel molecular signatures and pathogenesis in cutaneous T cell lymphoma

**DOI:** 10.1038/s41419-022-05323-5

**Published:** 2022-11-18

**Authors:** Xiaotong Xue, Zhenzhen Wang, Zihao Mi, Tingting Liu, Chuan Wang, Peidian Shi, Lele Sun, Yongliang Yang, Wenchao Li, Zhe Wang, Hong Liu, Furen Zhang

**Affiliations:** 1 Shandong Provincial Hospital for Skin Diseases & Shandong Provincial Institute of Dermatology and Venereology, Shandong First Medical University & Shandong Academy of Medical Sciences, Jinan, Shandong China; 2 Shandong Provincial Hospital Affiliated to Shandong First Medical University, Jinan, Shandong China

**Keywords:** Tumour heterogeneity, Oncogenesis

## Abstract

Sézary syndrome (SS) is a rare and aggressive type of cutaneous T cell lymphoma (CTCL) with a poor prognosis. Intra-tumoral heterogeneity caused by different disease compartments (e.g., skin, blood) and poor understanding of the pathogenesis has created obstacles to the precise diagnosis and targeted treatment of the disease. Here we performed a comprehensive analysis by integrating single-cell transcriptomic data of 40,333 peripheral blood mononuclear cells (PBMCs) and 41,580 skin cells, as well as single-cell chromatin accessibility data of 11,058 PBMCs from an SS patient and matched healthy controls (HCs). Validation and functional investigation were carried out in an independent cohort consisting of SS patients, mycosis fungoides (MF) patients, psoriatic erythroderma patients, and HCs, as well as multiple cell lines. The analysis revealed that skin-derived Sézary cells (SCs) had a shifting trend to more advanced mature phenotypes compared to blood-derived SCs. A series of specific marker genes (*TOX*, *DNM3*, *KLHL42*, *PGM2L1*, and *SESN3*) shared in blood- and skin-derived SCs were identified, facilitating the diagnosis and prognosis of MF/SS. Moreover, luciferase reporter assays and gene knockdown assays were used to verify that *KLHL42* was transcriptionally activated by *GATA3* in SS. Functional assays indicated that *KLHL42* silencing significantly inhibited aggressive CTCL cell proliferation and promoted its apoptosis. Therefore, targeting inhibition *KLHL42* might serve as a promising therapeutic approach in CTCL.

## Introduction

Sézary syndrome (SS) is a rare and aggressive CD4^+^ leukemic variant of cutaneous T cell lymphoma (CTCL) with a disease-specific 5-year survival of 36% and median survival of 2–4 years [1]. It is characterized by diffuse pruritic erythroderma, lymphadenopathy, and atypical clonal T cells (Sézary cells, SCs) accumulated in the peripheral blood (PB), skin, and lymph nodes [2]. Previously, SCs were thought to originate from mature, monoclonal, and central memory T cells (T_CM_) [3]. However, a great diversity of SCs has recently been reported in terms of naive/memory maturation phenotype and molecular signature [4]. There is some evidence that CTCL might derive from circulating immature precursor cells [5–7]. The origin and phenotype of SCs remain undetermined.

The lack of specific marker genes to accurately distinguish SCs and benign reactive T cells is a challenge for the early diagnosis of SS. A series of novel molecular biomarkers, such as *KIR3DL2* (*CD158k*), *PLS3*, *TWIST1*, and *NKp46* were considered for the diagnosis of SS. However, not a single one showed sufficient specificity and sensitivity due to inter- and intratrumoral heterogeneity [8]. Recently, single-cell RNA sequencing (scRNA-seq) of circulating SCs has shown that *FOXP3* along with another 19 genes can be used to predict CTCL stage with close to 80% accuracy [9]. Single-cell profiling of skin biopsies from advanced mycosis fungoides (MF)/SS patients has revealed a common gene expression signature (*PCNA*, *ATP5C1*, and *NUSPA1*) with important implications for diagnosis and treatment of CTCL, even though only a few tumor-specific marker genes were shared in different samples [10]. However, intra-tumoral heterogeneity within different disease compartments (e.g., skin, blood) further added complexity to SS, possibly creating obstacles for the diagnosis of the disease [11], which remains unexplored. Therefore, the identification of a body of diagnostic malignant cell markers, especially from different compartments, and their optimal combination have great potential in improving the diagnostic rate of SS and its early diagnosis.

Owing to the poor understanding of disease pathogenesis, effective treatments for SS are limited and have poor prognosis. Traditional and novel targeted therapies, including bexarotene, histone deacetylase inhibitors, anti-CCR4 antibody (mogamulizumab), and anti-CD52 antibody (alemtuzumab), did not achieve adequate complete response rates (10%-47%) in advanced-stage MF/SS [12–15]. Investigating the novel pathogenesis and finding new therapeutic targets for SS cells within different compartments would facilitate the treatment of SS. Epigenetics plays a central role in the pathogenesis of various cancers, including CTCL [16]. ScRNA-seq combined with the single-cell assay for transposase-accessible chromatin using sequencing (scATAC-seq) has been demonstrated to elucidate the disease-associated transcriptional regulation mechanisms in acute leukemia [17], providing new avenues to explore the pathogenesis of CTCL.

In this study, we performed single-cell transcriptomic analysis of skin biopsies and PBMCs from an SS patient and matched healthy controls (HCs) to further reveal intra-tumoral heterogeneity across disease compartments and identified specific marker genes shared by malignant CD4^+^ T cells from different compartments. Moreover, single-cell transposase-accessible chromatin data from PBMCs were integrated with single-cell transcriptomic data to elucidate the pathogenesis of CTCL, providing novel therapeutic targets for the precision treatment of CTCL.

## Materials and Methods

Detailed experimental methods and data analysis are provided in Supplementary methods.

## Results

### Study design

**Fig. 1:**
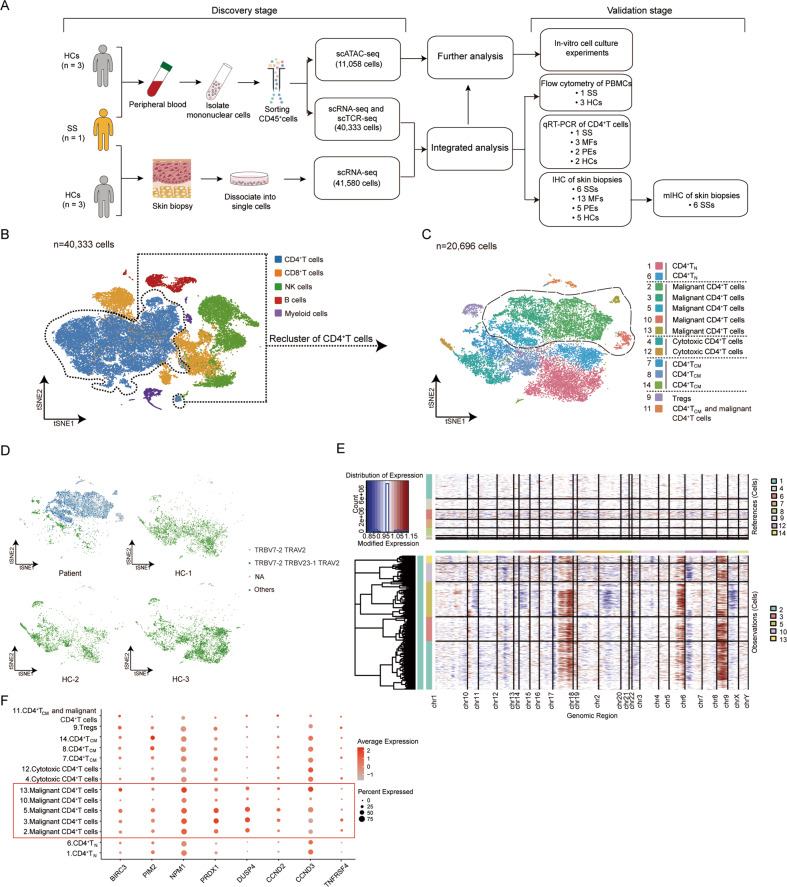
Identification and transcriptional characteristics of malignant CD4^+^ T cells in PBMCs. **A** Overview of study design. **B** t-SNE visualization of PBMCs scRNA-seq profile showing the formation of five main clusters. **C** Sub-clustering of CD4^+^ T cells in PBMCs from the SS patient and HCs, showing the formation of 14 main clusters. **D** TCR analysis of PBMC CD4^+^ T cells from the SS patient and HCs. **E** CNV analysis of PBMC CD4^+^ T cells from the SS patient and HCs. **F** Dot plot showing the expression of selected cell-cycle, proliferation, and T-cell activation-associated genes in each cluster of PBMC CD4^+^ T cells from the SS patient and HCs.

In the discovery stage, PBMCs from one SS patient and three HCs were collected to perform scRNA-seq, scTCR-seq, and scATAC-seq. Skin lesion from an SS patient and normal skin biopsies from three HCs were collected for scRNA-seq. For validation experiments, including flow cytometry (FC), immunohistochemistry (IHC), and quantitative real-time RT-PCR (qRT-PCR), clinical samples from 19 CTCL patients (six SS patients, five advanced-stage MF patients, and eight early-stage MF patients), seven psoriatic erythroderma (PE) patients, and 10 HCs were used. In vitro cell culture experiments were performed to further validate the results of integrated scRNA-seq and scATAC-seq data. The flow chart for the study design is represented in Fig. 1A.

### Identification and transcriptional characteristics of malignant CD4^+^ T cells in PBMCs

We acquired single-cell transcriptomes in a total of 40,333 CD45^+^ immune cells from PBMCs of one SS patient and three HCs. Five major cell types, including CD4^+^ T, CD8^+^ T, NK, B, and myeloid cells, were identified based on the expression of canonical gene markers (Figs. 1B, S1A). All of these cell subtypes were shared by the SS patient and HCs (Fig. S1B and Table S1A). An abnormal proportion of CD4^+^ T cells (88.72%) was observed in the SS patient compared to an average of 39.19% in the HCs (Fig. S1C and Table S1A).

We next performed re-clustering of CD4^+^ T cells from PBMCs of the SS patient and three HCs, identifying 14 clusters (Figs. 1C, S2A). Although all clusters contained cells from each donor, clusters 2, 3, 5, 10, and 13 were mostly from PBMCs of the SS patient (Fig. S2B-C and Table S1B). Consistent with neoplastic cells previously described in advanced CTCL, CD4^+^ T cells from these clusters showed a dominant clonotype (TRBV7-2 and TRAV2) and apparent copy number variations [9, 18] (Figs. 1D, E). They also exhibited an increased aberrant expression of tumor-associated genes *KIR3DL2* (*CD158k*) and *CD70*, and a decrease in *CD7* [9, 19] (Fig. S2A). In addition, these clusters corresponded to highly expressed genes involved in survival (e.g., *BIRC3*), proliferation (e.g., *PIM2*, *NPM1*, and *PRDX1*), cell cycle (e.g., *CCND2*, *CCND3*, and *DUSP4*) and T cell activation (e.g., *TNFRSF4*), further suggesting that CD4^+^ T cells from clusters 2, 3, 5, 10, and 13 represented high levels of proliferated and activated malignant CD4^+^ T cells [10, 20] (Fig. 1F). These five subsets presented a phenotype for T_CM_ cells (*SELL*^+^*CCR7*^+^*CD27*^+^*TCF7*^+^*S100A4*^+^) and were weakly positive for skin homing molecules *CCR4* and *CCR10* [21, 22] (Fig. S2A).

### Identification and transcriptional characteristics of malignant CD4^+^ T cells and tumor microenvironment in skin tissues

**Fig. 2:**
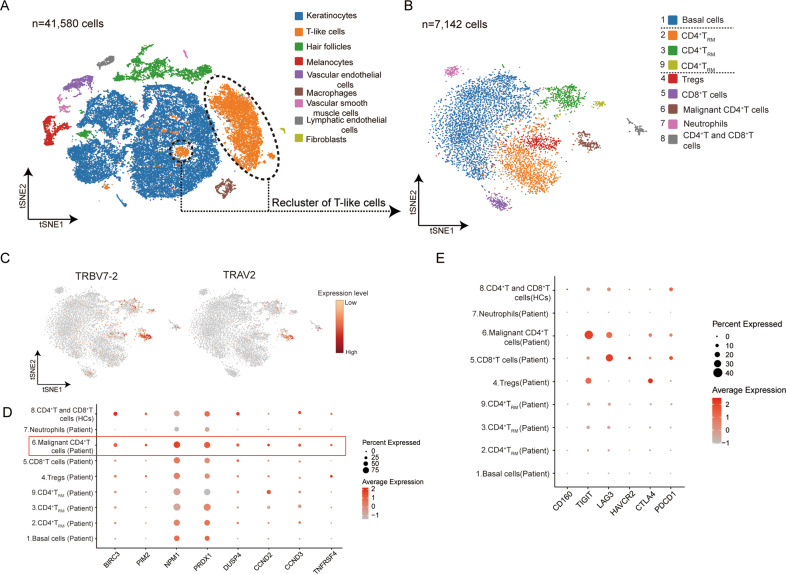
Identification and transcriptional characteristics of malignant CD4^+^ T cells and tumor microenvironment in skin tissues. **A** t-SNE visualization of skin scRNA-seq profile showing the formation of nine main clusters. **B** Sub-clustering of skin T-like cells from the SS patient and HCs, showing the formation of nine main clusters. **C** *TRBV7-2* and *TRAV2* showing higher frequency in cluster 6 from skin T-like cells in the SS patient. **D** Dot plot showing the expression of selected cell-cycle, proliferation, survival, and T-cell activation associated genes in each cluster of skin T-like cells from the SS patient and HCs. **E** Dot plot showing the proportion of cells expressed co-inhibitory receptors in each cluster of skin T-like cells from the SS patient and HCs.

A total of 41,580 qualified skin cells from an SS skin lesion and three normal skin biopsies were analyzed using scRNA-seq. Nine main cell types (keratinocytes, T-like cells, hair follicle cells, melanocytes, vascular endothelial cells, macrophages, lymphatic endothelial cells, vascular smooth muscle cells, and fibroblasts) were identified using canonical gene markers (Fig. 2A, Fig. S3A). All of these cell subtypes were shared by the four skin samples at different proportions, and the greatest heterogeneity between SS and controls was observed in T-like cells (average of 28.87% vs. 1.13%; Fig. S3B-C and Table S2A).

Further re-clustering of T-like cells from skin tissues of the SS patient and three HCs identified nine clusters (Figs. 2B, S4A and Table S2B). The high expression of clonotype genes (*TRBV7-2* and *TRAV2*) in cluster 6, the highest frequency of TCRα clonotype TRAV2 and TCRβ clonotype TRBV7-2 in the SS patient’s skin lesion, the increased aberrant expression of tumor-associated genes *KIR3DL2* (*CD158k*) and a decrease in *CD7* in cluster 6 indicated that cells in cluster 6 were mainly malignant CD4^+^ T cells [9, 19] (Figs. 2C, S4A-B). Benign lymphocytes were distributed in clusters 2, 3, 4, 5, and 9 from the SS patient, as well as cluster 8 from HCs (Fig. S4C). Consistent with blood-derived malignant CD4^+^ T cells, skin-derived malignant CD4^+^ T cells highly expressed a group of genes associated with T cell proliferation and activation [10, 20] (Fig. 2D). Malignant CD4^+^ T cells from skin were rich in *SELL*, *CCR7*, and *CD27*, showing characteristics of the T_CM_ phenotype, which was similar to the phenotype of circulating neoplastic cells (Fig. S4A). Moreover, cluster 6 expressed skin-homing molecule genes (*CCR4* and *CD69*) and tissue-resident associated gene *NR4A1* [22, 23], suggesting a T_RM_ phenotype (Fig. S4A).

We further analyzed the expression of inhibitory receptors in T cell clusters to understand the skin tumor microenvironment. High expression of multiple inhibitory receptors expressed in reactive CD8^+^ T cells, and *TIGIT* in Tregs prompted the immunosuppressive tumor microenvironment in SS [24, 25] (Fig. 2E). Targeting co-inhibitor receptors to reverse the immunosuppressive state might aid in reactivating anti-tumor immunity and tumor treatment. In addition, *TIGIT* and *LAG3* were highly expressed in malignant CD4^+^ T cells, as previously reported [26] (Fig. 2E). Further studies are needed to investigate the functional and biological significance of *TIGIT* and *LAG3* in SS.

### Transcriptional heterogeneity between blood- and skin-derived SCs

**Fig. 3:**
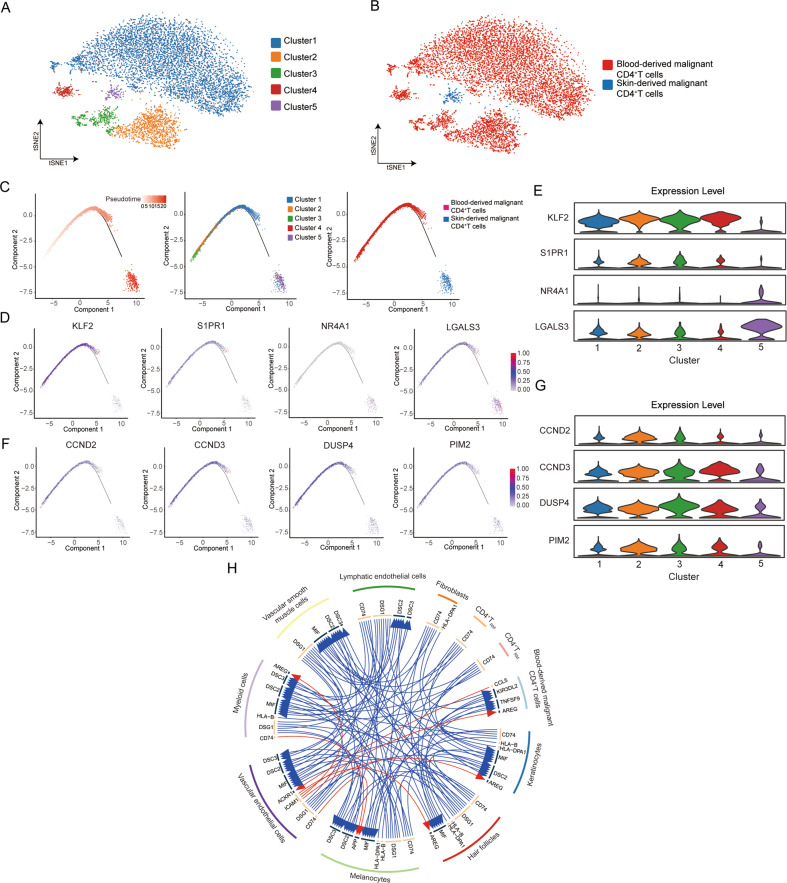
Transcriptional heterogeneity between blood- and skin-derived SCs. **A** t-SNE visualization of scRNA-seq data from blood- and skin-derived malignant CD4^+^ T cells in the SS patient. **B** The proportions of different compartments in five clusters. **C** Pseudotime trajectory of blood- and skin-derived malignant CD4^+^ T cells was generated by Monocle 2. **D** Trajectory plots with the expression of respective T-cell differentiation-associated genes, highest expression in red, lowest expression in grey. **E** Violin plot showing the expression of respective T-cell differentiation-associated genes in each cluster. **F** Trajectory plots with the expression of respective cell-cycle and proliferation-associated genes, highest expression in red, lowest expression in grey. **G** Violin plot showing the expression of respective cell-cycle and proliferation-associated genes in each cluster. **H** The interactions between malignant CD4^+^ T cells from PBMCs and all cell subtypes from skin. The strong interactions were indicated by red arrows.

To explore the differentiation relationship between blood- and skin-derived SCs in SS, transcriptional comparisons and pseudotime trajectory analysis following the pooling and clustering of malignant CD4^+^ T cells from peripheral blood (PB) and skin were performed (Fig. 3A). The results showed that skin-derived malignant CD4^+^ T cells were concentrated in cluster 5, while other four clusters mainly contained blood-derived malignant CD4^+^ T cells (Fig. 3B). Blood- and skin-derived malignant CD4^+^ T cells were distributed in the two trajectory termini (Fig. 3C). T-cell differentiation-associated genes were further assessed along the pseudotime trajectory, showing that the levels of transcription factor *KLF2* and its target gene *S1PR1* were significantly reduced in skin-derived malignant CD4^+^ T cells compared to blood-derived malignant CD4^+^ T cells, which have been reported as critical steps in the tissue retention and development of CD4^+^ T_RM_ cells [27] (Fig. 3D, E). The transcription factor *NR4A1*, which could influence the function and differentiation of T_RM_ cells, and surface molecule *LGALS3*, as a new marker for human skin T_RM_, were highly expressed in skin-derived malignant CD4^+^ T cells [23, 28] (Fig. 3D, E). In addition, the expression of cell cycle-associated genes, such as *CCND2*, *CCND3*, and *DUSP4*, and proliferation-associated genes, such as *PIM2*, significantly decreased during the transition from blood- to skin-derived malignant CD4^+^ T cells [10, 20] (Fig. 3F, G). Based on the phenotype of blood- and skin-derived SCs, skin-derived SCs could differentiate into cells with a more advanced maturation T_RM_ phenotype with low proliferation compared to blood-derived SCs. Moreover, cell-cell interactions between malignant CD4^+^ T cells from PB and all cell types from skin showed that blood-derived malignant CD4^+^ T cells strongly communicated with vascular endothelial cells via chemokine ligand-receptor axes, such as CCL5-ACKR1 and ICAM1-AREG axes [29, 30] (Fig. 3H), further illuminating the mechanism of blood-derived neoplastic cell homing to the skin.

### Identification of specific marker genes correlated with MF/SS disease progression

**Fig. 4:**
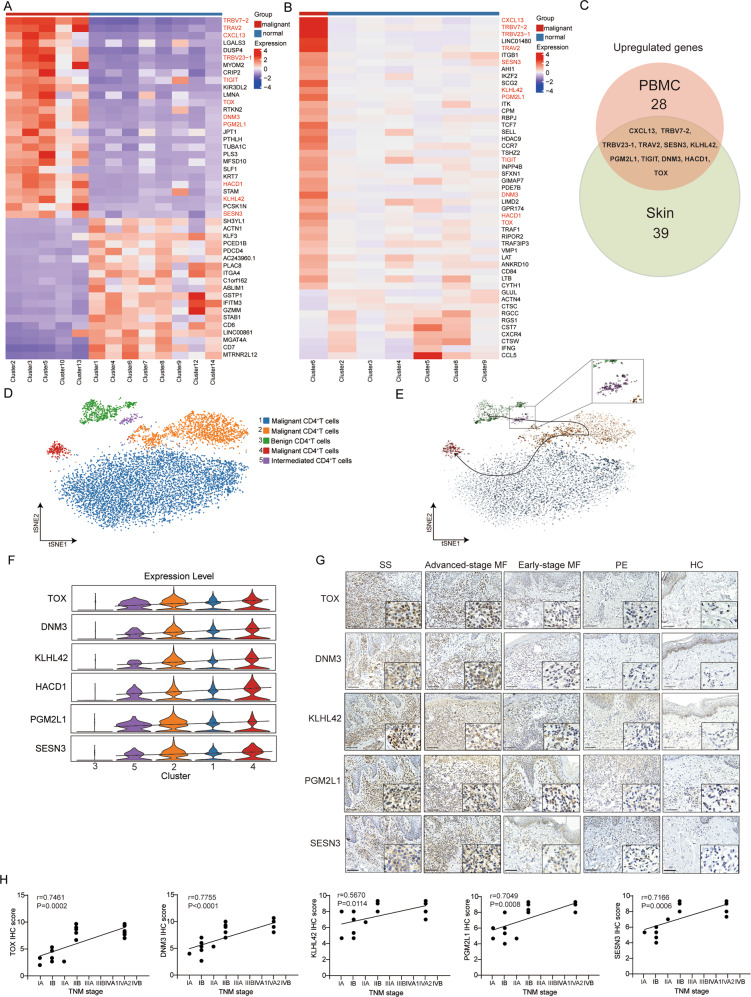
Identification of specific marker genes correlated with MF/SS disease progression. **A** Heatmap of significantly DEGs in each subset of malignant and benign CD4^+^ T cells from PBMCs. **B** Heatmap of significantly DEGs in each subset of malignant and benign lymphocytes from skin tissues. **C** Venn diagram showing overlap of significantly upregulated expressed genes of blood- and skin-derived malignant CD4^+^ T cells. **D** t-SNE visualization of scRNA-seq data from PBMC CD4^+^ T cells in the SS patient. **E** RNA velocity analysis of PBMC CD4^+^ T cells in the SS patient. **F** Violin plots showed the expression of *TOX*, *DNM3*, *KLHL42*, *HACD1*, *PGM2L1*, and *SESN3* in each cluster. **G** Immunohistochemical stain of *TOX*, *DNM3*, *KLHL42*, *PGM2L1*, and *SESN3* in skin biopsies of HC, PE patient, early-stage MF patients, advanced-stage MF patients and SS patients, each at 200× (left) and 400× (right). **H** Correlation analysis of disease stage with IHC scores of *TOX*, *DNM3*, *KLHL42*, *PGM2L1*, and *SESN3* in MF/SS. Pearson’s correlation coefficient.

In order to investigate potential specific markers of malignant CD4^+^ T cells, differential gene expression analysis was performed between malignant (clusters 2, 3, 5, 10, and 13) and benign (clusters 1, 4, 6, 7, 8, 9, 12, and 14) CD4^+^ T cells in PBMCs. The top 47 significant differential expressed genes (DEGs) were identified in malignant CD4^+^ T cells, including 28 up-regulated genes (specifically expressed in malignant CD4^+^ T cells) and 19 down-regulated genes (almost absent in malignant CD4^+^ T cells) (Fig. 4A, Table S3). Furthermore, differential gene expression analysis between malignant CD4^+^ T cells (cluster 6) and benign lymphocytes (clusters 2, 3, 4, 5, 8, and 9) in skin tissues identified 39 significantly up-regulated genes (specifically expressed in malignant CD4^+^T cells) and 10 significantly down-regulated genes (almost absent in malignant CD4^+^ T cells) (Fig. 4B, Table S4). In addition, an 11-gene expression signature (*CXCL13*, *SESN3*, *KLHL42*, *TRBV7-2*, *TRBV23-1*, *TRAV2*, *PGM2L1*, *TIGIT*, *DNM3*, *HACD1*, and *TOX*) was determined, such that the genes had a significantly high expression in both blood- and skin-derived malignant CD4^+^ T cells (Fig. 4C).

To further explore the relationship between the expression of the aforementioned genes and the malignant degree of circulating CD4^+^ T cells, we performed unsupervised clustering of all circulating CD4^+^ T cells from the SS patient (Fig. 4D). Combined with the results of gene expression signature and RNA velocity, malignant CD4^+^ T cells (*CD158k*^+^*CD70*^+^*TRBV7-2*^+^*TRAV2*^+^*CD7*^-^) from cluster 1, 2, and 4, benign CD4^+^ T cells (*CD158k*^-^*CD70*^-^*TRBV7-2*^-^*TRAV2*^-^*CD7*^+^) from cluster 3, and intermediate CD4^+^ T cells (*CD158k*^+^*CD70*^+^*TRBV7-2*^+^*TRAV2*^+^*CD7*^+^) from cluster 5 were identified [9, 19, 31] (Figs. 4E, S5A). The existence of intermediate CD4^+^ T cells from cluster 5 (CD3^+^CD4^+^CD45RO^+^CD7^+^CD158k^+^) in PB was further validated by FC in the SS patient, but not in the three HCs [32] (Fig S5B-C). The expression of eight genes (*CXCL13*, *SESN3*, *KLHL42*, *PGM2L1*, *TIGIT*, *DNM3*, *HACD1*, and *TOX*) that were significantly upregulated in both blood- and skin-derived malignant CD4^+^ T cells was analyzed according to the RNA velocity trend, excluding the three clonotype genes (*TRBV7-2*, *TRBV23-1*, and *TRAV2*). We found that the expression of six genes (*TOX*, *DNM3*, *KLHL42*, *HACD1*, *PGM2L1*, and *SESN3*) was gradually elevated with the malignant degree of circulating CD4^+^ T cells, but was almost undetectable in benign CD4^+^ T cells (Figs. 4F, S5D). qRT-PCR further showed that the expression of those genes was significantly increased in circulating CD4^+^ T cells (> 80% neoplasm cells) from the SS patient compared to those from advanced-stage MF patients, PE patients, and HCs (Fig. S5E).

We then used IHC experiments to validate the expression of these genes in skin biopsies from MF/SS patients, showing that the expression of *TOX*, *DNM3*, *KLHL42*, *PGM2L1*, and *SESN3* was significantly increased in the dermis of SS, advantage-stage MF, and early-stage MF patients compared to that in PE patients and HCs (Figs. 4G, S6A). Multiple immunohistochemistry (mIHC) analysis demonstrated that most TOX-, DNM3-, KLHL42-, PGM2L1-, and SESN3-positive cells co-localized with CD4 in SS skin lesions (Fig. S6B). In addition, TOX-, DNM3-, KLHL42-, PGM2L1-, and SESN3-positive cells from advanced-stage MF patients were significantly increased and had a stronger staining compared to the dermis of early-stage MF patients (Figs. 4G, S6A). The expression of *TOX*, *DNM3*, *KLHL42*, *PGM2L1*, and *SESN3* showed no significant difference between advanced-stage MF and SS patients (Figs. 4G, S6A). Pearson correlation analysis demonstrated that the expression of these genes was positively correlated with the disease stage of MF/SS (Fig. 4H). Moreover, Kaplan-Meier analysis of 22 MF patients indicated that overexpression of *TOX*, *DNM3*, and *KLHL42* was negatively correlated with disease-specific survival rate in MF (Fig. S6C-E). Collectively, *TOX*, *DNM3*, *KLHL42*, *PGM2L1*, and *SESN3* might be a common gene expression signature in malignant CD4^+^ T cells from advanced-stage MF/SS, and could be developed as markers for its diagnosis and prognosis.

### ScATAC-seq revealed *KLHL42*-associated transcriptional regulation mechanism in malignant CD4^+^ T cells

**Fig. 5:**
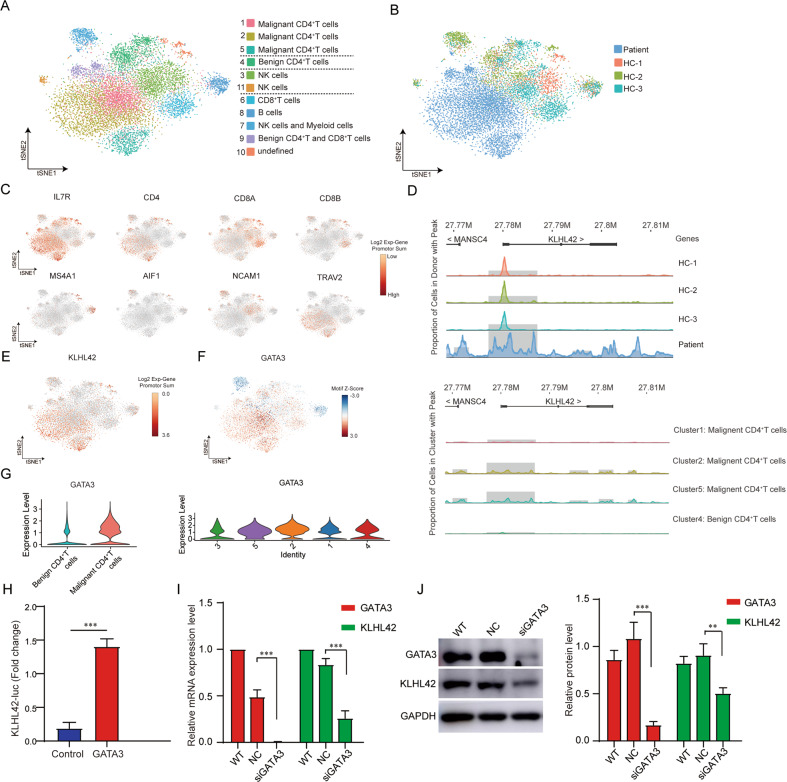
ScATAC-seq revealed *KLHL42*-associated transcriptional regulatory mechanism in malignant CD4^+^ T cells. **A** t-SNE projection of scATAC-seq profiles from PBMCs of the SS patient and three HCs. **B** t-SNE plot for PBMC CD4^+^ T cells split by the SS patient and HCs. **C** Expression of marker genes for major cell types. **D** Genome tracks of scATAC-seq data at the *KLHL42* locus in the SS patient and HCs. And genome tracks of scATAC-seq data at the *KLHL42* locus in benign CD4^+^ T cells (Cluster 4) and malignant CD4^+^ T cells (Cluster1, 2, 5). Highlighted regions showing cell type-specific ATAC-seq peaks. **E** t-SNE projection colored by log2 Exp-gene promoter sum demonstrating the chromatin accessibility of *KLHL42*. **F** t-SNE projection colored by chromVAR deviation z-score of TF motif demonstrating the acvivity of *GATA3*. **G** The former showing the expression of *GATA3* in benign (clusters 1, 4, 6, 7, 8, 9, 12, 14) and malignant (clusters 2, 3, 5, 10, 13) CD4^+^ T cells, clustering referred to Fig. 1C. And the later showing the expression of *GATA3* in benign (cluster 3), intermediate (cluster 5), and malignant (clusters 1, 2, 4) CD4^+^ T cells, clustering referred to Fig. 4D. **H** Dual-luciferase reporter assays showing that *GATA3* overexpression increased the luciferase activity in HEK293T cells transfected with the *KLHL42* promoter plasmid. **I**, **J** The mRNA and protein levels of *GATA3* and *KLHL42* in Hela cells with or without *GATA3* knockdown were measured by qRT-PCR (**I**) and western blotting (**J**). WT, wild type; NC, negative control. Data are presented as mean ± s.d. **P < 0.01, ***P < 0.001.

To further investigate the mechanisms that regulating the expression of the aforementioned genes, we performed scATAC-seq for 11,058 CD45^+^ immune cells in PBMCs from the SS patient and three HCs to analyze chromatin accessibility and TF-binding motifs (Fig. 5A, B, Table S5). Malignant CD4^+^ T cell subsets, including clusters 1, 2, 4, and 5, were identified based on high accessibility of *IL7R*, *CD4*, and *TRAV2* (Fig. 5C). Cluster 4 mainly consisted of benign CD4^+^ T cells with high accessibility of *IL7R* and *CD4*, as well as lower *TRAV2* accessibility (Fig. 5C).

Chromatin accessibility status of the biomarker genes, including *TOX*, *DNM3*, *KLHL42*, *PGM2L1*, and *SESN3*, was analyzed in PBMCs from the SS patient and HCs. The results showed a higher chromatin accessibility of *KLHL42* in malignant CD4^+^ T cells (clusters 1, 2, and 5) from the SS patient than in benign CD4^+^ T cells (cluster 4) from HCs (Fig. 5D, E), indicating that the *KLHL42* expression was regulated by epigenetic modification. Chromatin accessibility of the other four biomarker genes had no significant differences between malignant CD4^+^ T cells and benign immune cells (Fig. S7A). Based on the JASPAR and UCSC databases, we predicted transcription factors (TFs) that could bind to the *KLHL42* promoter (Fig. S7B). Further TF motif analysis showed that the *GATA3* motif was differentially enriched in malignant CD4^+^ T cells but deficient in benign immune cells (Fig. 5F). Meanwhile, *GATA3* mRNA expression was significantly increased in malignant CD4^+^ T cells compared to benign CD4^+^ T cells (Fig. 5G), suggesting that *GATA3* positively regulated the expression of *KLHL42* in malignant CD4^+^ T cells. Luciferase reporter gene assay showed that *GATA3* expression significantly enhanced the transcription activity of *KLHL42* promoter, while *GATA3* knockdown significantly suppressed the mRNA and protein expression of *KLHL42*, confirming the activation of *KLHL42* transcription by *GATA3* (Fig. 5H–J). Collectively, transcriptional activation of *KLHL42* by *GATA3* further upregulated *KLHL42* in malignant CD4^+^ T cells when chromatin *KLHL42* accessibility was high.

### *KLHL42* knockdown inhibited CTCL cell proliferation and promoted its apoptosis in vitro

**Fig. 6:**
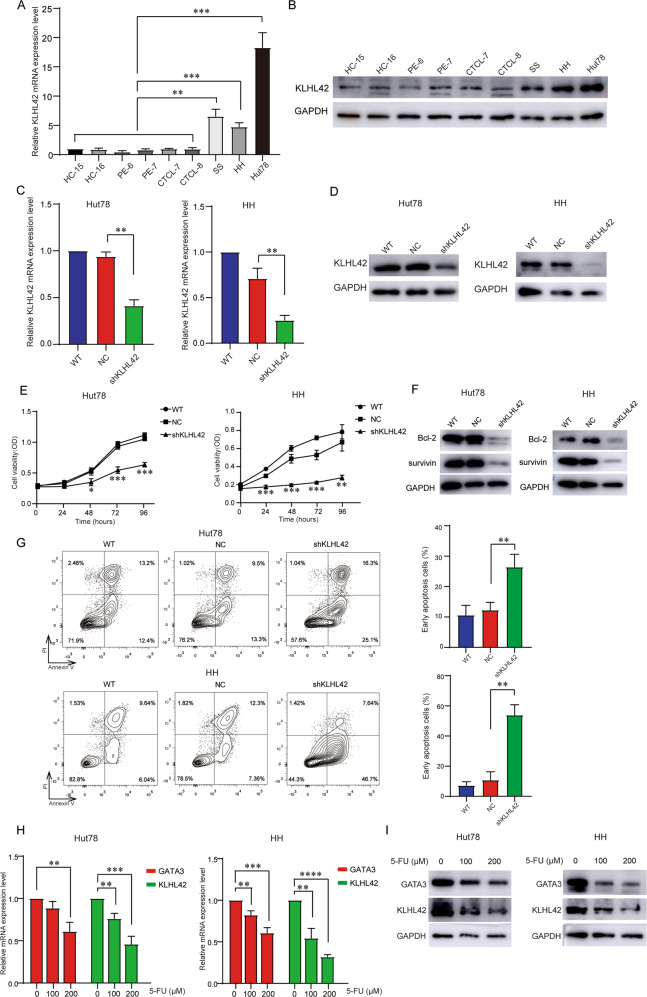
*KLHL42* knockdown inhibited CTCL cell proliferation and promoted its apoptosis in vitro. **A**, **B** The *KLHL42* mRNA and protein expression of PBMC CD4^+^ T cells in two HCs, two PE patients, two advanced-stage MF patients and one SS patient as well as HH and Hut78 cells were measured by qRT-PCR (**A**) and western blotting (**B**). **C**, **D** Suppression of *KLHL42* mRNA and protein expression in Hut78 and HH cells by lentiviral transduction with shRNA sequences. **E** CCK-8 proliferation assays in Hut78 and HH cells with or without *KLHL42* silencing. **F** Western blotting analysis of anti-apoptosis proteins in Hut78 and HH cells with or without *KLHL42* silencing. **G** Long-term culture of the transduced cells revealed an increase in the Annexin V^+^PI^-^ (early apoptosis) population in *KLHL42*-silenced cells compared with control cells. **H**, **I** 5-FU repressed *GATA3* and *KLHL42* expression in a dose-dependent manner. The mRNA and protein levels of both *GATA3* and *KLHL42* in Hut78 and HH cells were measured by qRT-PCR and western blotting. WT, wild type; NC, negative control. Data are presented as mean ± s.d. *P < 0.05, **P < 0.01, ***P < 0.001, ****P < 0.0001.

qRT-PCR and western blotting were performed to evaluate the expression of *KLHL42* and showed a significantly higher expression of *KLHL42* in CD4^+^ T cells from the SS patient (mainly malignant cells), HH cells (derived from a late-stage aggressive MF patient), and Hut78 cells (derived from a Sézary patient) compared to CD4^+^ T cells from advanced-stage MF patients, PE patients, and HCs [33] (Fig. 6A, B). To further confirm the function of *KLHL42* in CTCL, *KLHL42* expression was silenced in Hut78 and HH cells via lentivirus-mediated transduction (Figs. 6C, D, S8A). CCK-8 assays demonstrated decreased cell growth rates in *KLHL42*-silenced Hut78 and HH cells compared to the control cells (Fig. 6E). Moreover, the expression of anti-apoptosis proteins, such as Bcl-2 and survivin, was suppressed by *KLHL42* silencing (Figs. 6F, S8B). Annexin V-PI staining showed increased spontaneous apoptosis in *KLHL42*-silenced cells compared to the control cells (Fig. 6G). These results indicated that *KLHL42* knockdown inhibited Hut78 and HH cell proliferation and induced their apoptosis. The 5-fluorouracil (5-FU) is a chemotherapeutical agent previously reported to treat advanced-stage CTCL [34]. A recent study indicated that 5-FU effectively inhibited the expression of *KLHL22* [35]. We found that 5-FU repressed the mRNA and protein expression of both *GATA3* and *KLHL42* in a dose-dependent manner (Figs. 6H, I, S8C). In addition, 5-FU inhibited Hut78 and HH cell viability and induced their apoptosis (Fig. S9A-D). However, 5-FU didn’t significantly repress the expression of other specific marker genes (*TOX*, *DNM3*, *PGM2L1*, and *SESN3*) (Fig. S9E-F). Therefore, specific repression of *KLHL42* by *GATA3* might be one of the critical routes for 5-FU preventing tumor progression in CTCL. Collectively, the aberrant expression of *KLHL42* might play an important role in disease progression of CTCL, and targeting *KLHL42* inhibition might serve as a promising therapeutic approach for CTCL treatment.

## Discussion

CTCLs including MF and SS, have a striking clinical, biological, and molecular feature heterogeneity which presents challenges for tumor diagnosis and treatment [36]. Inter- and intra-tumoral heterogeneity in SS has been described by recent single-cell studies [9, 10]. In addition, variable microenvironments across disease compartments (e.g., skin, blood) might further complicate tumor heterogeneity [20]. Finding specific marker genes that target SCs within different compartments could improve the diagnosis, prognosis, and therapy of lymphoma. Here we analyzed single-cell transcriptomic profiles of PBMCs and skin cells from an SS patient and matched HCs, showing transcriptional heterogeneity and phenotypic plasticity of SCs across different tissues. A series of potential biomarker genes shared with blood- and skin-derived SCs were identified, including reported (*TOX*, *DNM3*, and *KLHL42*) and novel (*PGM2L1* and *SESN3*) tumor-associated genes, which could help to differentiate between SS and erythrodermic inflammatory dermatoses (EIDs), and facilitate the diagnosis and prognosis of MF/SS. Based on these and scATAC-seq data, a novel mechanism for *KLHL42*-associated transcriptional regulation was described, further elucidating the pathogenesis of CTCL and providing novel therapeutic targets for precise treatment of the disease.

SCs have been previously considered to originate from T_CM_ cells with a high expression of skin addressing (*CCR4*, *CCR10*, and *CLA*) [3]. Recent studies have found that some SCs from PB and skin presented as naive T (T_N_), stem-cell memory T (T_SCM_), and effector memory T (T_EM_) cell phenotypes, indicating the great diversity of SCs [4, 37]. Skin-derived SCs showed a more advanced maturation pattern than do their blood-derived compartment [4]. The present study found that blood-derived SC subsets presented with a T_CM_ phenotype, and skin-derived SCs showed the characteristics of T_CM_ and T_RM_ phenotype. Skin-derived SCs were accompanied by upregulation of *NR4A1* and *LGALS3*, as well as downregulation of *KLF2* and *S1PR1*, which aided in tissue residence and differentiation of T_RM_ [23, 27, 28]. In addition, blood-derived SCs showed a greater proliferation than skin-derived SCs. Blood- and skin-derived SCs presented the same dominant clone, indicating that the phenotypic heterogeneity of SCs was caused by their adaptive plasticity to different tissue microenvironment, rather than clonal heterogeneity [5]. Combined with the interaction between blood-derived SCs and cutaneous vascular endothelial cells, the present results suggested the existence of cases with an adaptive trend from a recirculating T_CM_ phenotype with active proliferation to a more advanced mature T_RM_ phenotype in the process of SC skin homing.

Reliable biomarkers can aid in the precise diagnosis and treatment of the disease. Intra-tumoral heterogeneity across compartments might create obstacles in identifying different tissue-derived SCs [38]. Identification of common gene expression SC signatures, especially from different compartments, could facilitate the diagnosis and treatment of SS. The present study identified five potential biomarker genes (*TOX*, *DNM3*, *KLHL42*, *PGM2L1*, and *SESN3*) that were specifically overexpressed in both blood- and skin-derived SCs and could help to distinguish between SS and PE patients. These genes were also highly expressed in MF patients compared to PE patients and HCs. The expression of these genes was positively associated with the disease progression of MF/SS. In addition, overexpression of *TOX*, *DNM3*, and *KLHL42* correlated with poor overall survival in MF patients. Previously, the high expression of *TOX* in skin and blood of SS patients has been reported to distinguish SS from other erythrodermic diseases. *TOX* had an oncogenic role in CTCL, supporting CTCL cell proliferation and survival [39, 40]. *DNM3*, which encodes microtubule-associated force-producing protein, and *KLHL42*, which encodes a substrate-specific adaptor protein of BTB-CUL3-RBX1 (BCR) E3 protein ligase, also have been reported to be highly expressed in SCs [41, 42]. The overexpression of *PGM2L1*, a glycolysis-related gene, has been reported to be associated with poor prognosis of prostate cancer patients with biochemical recurrence [43]. *SESN3*, which is an encoding member of the sestrin family of stress-induced proteins, served as an oncogene in esophageal squamous cell carcinoma cells [44]. *PGM2L1* and *SESN3* were shown to be overexpressed in SS patients and advanced-stage MF patients for the first time in the present study. Collectively, these genes could act as potential specific marker genes of malignant CD4^+^ T cells, facilitating the diagnosis and prognosis of MF/SS, as well as differentiation between SS and EID.

Dysregulation of TFs and epigenetic modifications play an important role in tumor progression [45]. In the present study, *KLHL42* overexpression in malignant CD4^+^ T cells corresponded to high accessibility of the *KLHL42* region, indicating that *KLHL42* expression was subject to the impact of certain epigenetic modifications. In addition, *GATA3*, the master regulator of Th2 CD4^+^ T cells [46], was significantly upregulated in malignant CD4^+^ T cells, and further upregulated the expression of *KLHL42* by activating the *KLHL42* promoter. Kelch-like family members are involved in ubiquitination regulation and might play a role in the progression of multiple tumors [47]. Deregulated cell proliferation, together with the obligate compensatory suppression of apoptosis, is necessary to support neoplastic progression [48]. Our further investigation revealed that *KLHL42* downregulation inhibited Hut78 and HH cell proliferation and promoted its apoptosis and indicating that *KLHL42* might be an oncogene in CTCL. Previous studies found that 5-FU, combined with methotrexate, could effectively treat advanced MF/SS [34], but the specific molecular mechanism was unclear. Our study indicated that repressing of *GATA3*/*KLHL42* axis might be one critical function route of 5-FU inhibiting tumor progression. Collectively, these data described a novel pathogenesis where *GATA3* overexpression further positively regulated *KLHL42* expression and promoted tumor progression in CTCL when *KLHL42* chromatin accessibility was high. Targeting inhibition *KLHL42* might serve as a promising therapeutic approach for CTCL.

The study limitation of included the cohort of only one SS patient and matched HCs in the discovery stage. However, skin and PB samples from six SS patients, 13 MF patients, seven PE patients, and 10 HCs, as well as multiple cell lines, were used to conduct various validation experiments to further verify the findings for the SC-specific marker genes and *KLHL42*-associated pathogenesis in CTCL.

In conclusion, the present study revealed the transcriptional heterogeneity within SCs across different tissues and described the phenotypic plasticity of blood- and skin-derived SCs. A series of specific marker genes of SCs were identified in order to facilitate the diagnosis and prognosis of MF/SS, further illustrating the *KLHL42*-associated pathogenesis of CTCL and providing new insight for precision-targeted CTCL therapy.

## Supplementary information

Supplementary methods

Supplementary figures 1-9 and figure legends

Supplementary tables 1-9

Original Data File

Reproducibility Checklist

## Data Availability

All data used in this study have been deposited in the Genome Sequence Archive in BIG Data Center, Beijing Institute of Genomics, Chinese Academy of Sciences under the accession numbers of HRA000847, HRA000826, and HRA000145.
