# Supplementary material for: Single-cell analyses reveal novel molecular signatures and pathogenesis in cutaneous T cell lymphoma
Source: Cell Death Dis. 2022 Nov 18;13(11):970. doi: 10.1038/s41419-022-05323-5 (PMC9674677; doi:10.1038/s41419-022-05323-5)
Supplement: Supplementary file 1 — Supplementary methods [file 41419_2022_5323_MOESM1_ESM.docx]

**Supplementary methods**

**Human specimens**

All samples from six Sézary syndrome (SS) patients, 13 mycosis fungoides (MF) patients, seven psoriatic erythroderma (PE) patients, and 16 healthy controls (HCs) were collected at the Shandong Provincial Hospital for Skin Disease, Shandong First Medical University. Detailed demographic and clinical information were described in Supplementary Table S6. Cutaneous T cell lymphoma (CTCL) patients including MF and SS were diagnosed based on clinical manifestations, histopathological examination, and other auxiliary examinations, including TCR rearrangement and flow cytometry (FC) by dermatologists, and staged according to the most recent consensus [1]. PE patients were diagnosed based on clinical manifestations and histopathological examination. Control peripheral blood mononuclear cell (PBMC) samples were provided by healthy donors, and control skin biopsies were obtained from fracture patients without tumor-related disease. Participants gave written informed consent. Human research protocols were approved by the Institutional Review Board, Shandong Provincial Hospital for Skin Diseases. This study was conducted in accordance with the Declaration of Helsinki.

**Single-cell processing**

Density gradient centrifugation was used to isolate PBMCs using a lymphocyte separation solution (MD pacific). Before the barcode labeling of single cells, CD45^+^ immune cells from PBMCs were sorted using flow cytometry (BD Bioscience).

Skin biopsy specimens were disassociated with Dispase II (Sigma) to separate the epidermis and dermis. The minced epidermis was further digested with 0.25% Trypsin-EDTA (Gibco) for 30 min and filtered with a 70 μm cell strainer (Falcon). The dermis was digested with 1 mg/mL Collagenase P (Sigma-Aldrich) and 100 μg/mL DNase I (Sigma-Aldrich) for 50 min and filtered using a 70 μm cell strainer (Falcon).

**Single-cell RNA and TCR sequencing**

The single-cell TCR enrichment library and 5´ expression library were prepared according to the standard manufacturer’s protocol from 10xGenomics Single Cell 5’ v1 Reagent Kit (10xGenomics). A total of 4,000-24,000 cells were targeted for each sample. Amplified cDNA and final libraries were evaluated on an Agilent Bio-Analyzer using a High Sensitivity DNA Kit (Agilent Technologies). The libraries were then pooled and sequenced on NovaSeq 6000 (Illumina) at a depth of approximately 350 M-1,200 M reads per sample in order to ensure that each cell has an average of approximately 50,000 read pairs.

**Single-cell ATAC-seq**

Single-cell ATAC-seq targeting about 10,000 cells per sample was performed using Chromium Single Cell ATAC Library & Gel Bead Kit v1.1 (10xGenomics, 1000175). Each sample library was uniquely barcoded and quantified by PCR using a PhiX Control v3 (Illumina, FC-110-3001) standard curve. Libraries were then pooled and loaded on NovaSeq 6000 (Illumina) and sequenced to 25,000 unique reads per cell on average.

**Flow cytometry for PBMCs**

To analyze the ratio of intermediate CD4^+^ T cells in PBMCs from one SS patient and three HCs, PBMCs were harvested and stained with the indicated mAbs (anti-human CD3, CD4, CD45RO, CD7, and CD158k). Samples were acquired using the LSR-II analyzer. Detailed information on the antibodies is provided in Supplementary Table S7.

**Immunohistochemistry (IHC) and multiple IHC (mIHC)**

Paraffin-embedded tissue microarray was dewaxed and rehydrated to retrieve the antigens. The sections were incubated with primary antibodies at 4℃ overnight. After washing with PBS, the horse radish peroxidase (HRP)-conjugated secondary antibody was added and further incubated for 2h. The diaminobenzidine (Beyotime) kit was used to visualize antibody binding. After washing, nuclei were stained with hematoxylin. For mIHC, incubation with secondary antibodies and color development were performed using a four-color multiple fluorescent immunohistochemical staining kit (Absin) in accordance with the manufacturer’s instructions. The sections were imaged using the EVOSTM FL 377 Auto 2 Imaging System (Thermo Fisher Scientific). The antibodies against TOX, DNM3, KLHL42, PGM2L1, and SESN3, as well as HRP-conjugated goat anti-rabbit secondary antibody were used for IHC. The [above-mentioned](../../../../../../LenovoSoftstore/Install/wangyiyoudaocidian/8.9.9.0/resultui/html/index.html#/javascript:;) antibodies and CD4 antibody were used for mIHC. Detailed information on the antibodies is listed in Supplementary Table S7. The scoring system details are described in Supplementary Table S8. Three different fields of view (×200 magnification) in each slide were analyzed, and the mean value of the measurements was recorded by three observers as the final result.

**Cell cultures and transfection**

The cutaneous T cell lymphoma cell line Hut78 (derived from a Sézary patient) and HH (derived from a late-stage aggressive MF patient), human embryonic kidney cell line HEK293T, and Hela cell line were purchased from American Type Culture Collection (ATCC). Hut78 and HH cell lines are cultured used by RPM-I1640 medium supplemented with 10% fetal bovine serum and penicillin-streptomycin solution (100 units/mL) at 37℃ in a humidified atmosphere with 5% CO_2_. HEK293T and Hela cell lines are cultured used by DMEM medium supplemented with 10% fetal bovine serum and penicillin-streptomycin solution (100 units/mL) at 37℃ in a humidified atmosphere with 5% CO_2_. All cell lines were authenticated using STR profiling and verified as mycoplasma-free every month. The shRNA-KLHL42 and the negative control were purchased from Shanghai Genechem. The siGATA3 and the negative control were purchased from Shanghai GenePharma.

**Quantitative real-time RT-PCR (qRT-PCR)**

The total RNA was isolated by using the Trizol reagent (Invitrogen) followed the common protocol. Two micrograms of the total RNA of each sample was reverse transcribed by using the Ultrapure RNA Kit (CWBIO) according to the manufacturer’s instructions. Quantitative real-time PCR was performed on an FTC-3000 Real-Time PCR System. The SYBR Green Supermix was used for all of the real-time PCR assays. The thermal cycling conditions for quantitative real-time PCR including an initial denaturation step of 95℃ for 10s followed by 40 PCR cycles of 95℃ for 5s, 60℃ for 30s, and 72℃ for 10min. The reaction is terminated by chilling the mixture to 4℃. All samples were assayed in triplicate in each experiment. The relative amount of mRNA was calculated by using the comparative CT method after normalization to the internal control GAPDH mRNA levels. The sequence of primer for the target genes were as follows:

F: 5’-ACCACAACCACACTCTGGAGGA-3’

R: 5’-TCGGTTTCTGGTCTGGATGCCT-3’ for GATA3

F: 5’-CCAATCCTCTGGCTGAGTTCTC-3’

R: 5’-CAAAGAGCGTCCACATGTCGGA-3’ for KLHL42

F: 5’-CGCTACCTTTGGCGAAGTCTCT-3’

R: 5’-CTGGCTCTGTATGCTGCGAGTT-3’ for TOX

F: 5’-GCTCACCATCAGCAACATTGGC-3’

R: 5’-CCGAACTTTCAGGTTGTCCAAGG-3’ for DNM3

F: 5’-CTAGCCACAGATCCTGATGCAG-3’

R: 5’-CCACCATCCAAACAAAGCTGCC-3’ for PGM2L1

F: 5’-GACAGTGACCTGCTATCCTGAG-3’

R: 5’-CCGAGTTATGGCACGAAGAGCA-3’ for SESN3

F: 5’-TGGTGTGGCTCATTACTCACAG-3’

R: 5’-TGGCAAGTGGTCAAGAAGGC-3’ for HACD1

F: 5’-GTCTCCTCTGACTTCAACAGCG-3’

R: 5’-ACCACCCTGTTGCTGTAGCCAA-3’ for GAPDH

**Western blotting**

Cells were washed with Phosphate-Buffered Saline (PBS, 1×). Cells were lysed with RIPA buffer (140 mM NaCl, 10 mM Tris·Cl, 1% Triton X-100, 0.1% sodium deoxycholate, 0.1% SDS, 1 mM PMSF, plus protease inhibitor mixture, pH 8.0). Cell lysates equal to total protein 30 μg from each sample were subjected to SDS-PAGE (10% acrylamide). After SDS-PAGE, the proteins were transferred to PVDF membranes. Membranes were blocked with Tris-base buffer saline Plus tween 20 (TBST) containing 5% nonfat milk and 0.1% Tween-20 for 1 hr at room temperature. Primary antibodies were incubated for 2 hrs at room temperature, followed by multiple washes in 1×TBST. The appropriate secondary antibodies were incubated in the blocking solution for 1 hr at room temperature, followed by multiple washes with TBST. Chemiluminescence was detected using a SuperSignal West Femto maximum sensitivity substrate kit (Pierce) in accordance with the manufacturer's protocol. Rabbit polyclonal anti-KLHL42 antibody, anti-GATA3 antibody, anti-Bcl-2 antibody, anti-survivin antibody, anti-GAPDH antibody and goat anti-rabbit IgG secondary antibodies were used in western blotting. All of the detailed information on the antibodies was listed in Supplementary Table S7.

**Luciferase reporter gene assay**

Luciferase activities were measured with a Dual-Luciferase Reporter Assay System (Promega) according to the manufacturer’s instructions. Briefly, HEK293T cells were seeded into 24-well plates and 100 ng luciferase reporter (KLHL42-Luc) were co-transfected with 500 ng GATA3 expression vector or negative control plasmid. After 24h post-transfection, the lysed samples were prepared, and luciferase activity was detected by the DLR Assay System (Promega). Data were normalized for transfection efficiency by dividing the Firefly luciferase activity by that of Rlla luciferase.

**CCK-8 assay**

Hut78 and HH cells, dividing into WT, NC, and shKLHL42 groups, were plated in a 96-well plate at a concentration of 10^4^ cells per well. For cell proliferation assay, 10 μL of CCK-8 solution (Solarbio) was added to each well, and incubated at 37℃ for 1 hrs. The absorbance was measured at a wave length of 450 nm by using the microplate reader (Synergy 2 Multi-Mode Microplate Reader; BioTek).

**Apoptosis analysis**

Long-time culture of WT, NC, and shKLHL42 cells, as well as cells treated with 0μM, 100 μM, and 200 μM of 5-FU for 48 hours were stained with FITC Annexin V Apoptosis Detection Kit I (BD Pharmingen) and quantified by flow cytometry analysis using FACSCalibur (BD Biosciences). Data were analyzed with FlowJo 10.7.2 software.

**Analytical methods**

**Single-Cell RNA-seq Data Processing and Quality Control (QC)**

PBMCs and skin samples dataset were processed and quality controlled separately. For each samples dataset, raw sequencing data were aligned and quantified using the CellRanger pipeline (version 3.0.1, 10xGenomics) to the GRCH38 human reference genome. The gene expression matrix was then processed and analyzed by Seurat (version 3.2.0) R (version 3.6.3) [2]. For quality control, we removed the cells with total UMI (Unique Molecular Identifier) numbers fewer than 500 or lower than 200 genes or higher than 10000 genes. We also removed all cells with the proportion of mitochondrial gene counts higher than 10% or cells with more than 10% red cell reads. Cells that were predicted to be potential cell doublets by Python Scrublet package (version v0.2) were also excluded [3].

**Dimensionality Reduction, Clustering and Visualization**

For each sample dataset, the filtered expression matrix was first normalized using Seurat’s “NormalizeData” function with default parameters. The top 2000 highly variable Genes (HVGs) were identified through the function “FindVariableGenes”. Then those HVGs were used for the subsequent Principal Component Analysis (PCA) to reduce dimensionality by “RunPCA” function. Clusters were calculated by the “FindClusters” function with optimal resolution on the top 30 principal components. Visualization was achieved by the t-Distributed Stochastic Neighbor Embedding (t-SNE) with Seurat function “RunTSNE”. Finally, specific markers in each cluster were identified by the “FindAllMarkers” function and genes detected in at least 25% cells in the cluster with adjusted P values < 0.05 and log2 (foldchange) (log2FC) > 0.25 were considered as marker genes for the cluster. Clusters were assigned to known cell types based on the expression of canonical gene markers, previously identified markers and top 10 upregulated genes of each cluster. Subclustering for major cell types was performed in the same way to identify finer clusters. During re-clustering for skin T-like cell subsets, we observed some clusters of cells expressing keratinocyte markers such as KRT1 and SPRRB1 or neutrophils markers such as PI3, and we removed the clusters from further analysis.

Differentially expressed genes (DEGs) analysis between malignant and benign CD4^+^ T cells from PBMCs were performed using Likelihood-ratio test method of “FindMarkers” function and then |log2FC| > 0.25 and adjusted P values < 0.05 were considered significant. After excluding ribosomal associated genes, we screened top50 genes for further analysis based on the sort of |log2FC| from largest to smallest.

**TCR analysis**

The TCR sequences for each single T cell were assembled by the CellRanger vdj pipeline (v.3.1.0) to identify the CDR3 sequence and the rearranged TCR genes. We kept cells with both TCR alpha- and beta-chains.

**Pseudo-time lineage trajectory and RNA velocity**

Lineage trajectory plot based on variant feature identified by Seurat v3 was generated by monocle R package. To calculate the RNA velocities, we first annotated spliced/unspliced reads using CellRanger (version 2.2.0) and generated BAM files and an accompanying GTF. Then, we calculated the direction of differentiation trajectory of each cell and mapping RNA velocity vector to low-dimension space according to the scVelo python pipeline [4, 5].

**CNV analysis**

CNV score for each cells were estimated by the expression levels of genes within each chromosome region using inferCNV R package [6]. Clusters 1, 4, 6, 7, 8, 9, 12, and 14 (the normal cells from PBMCs) was used as the reference for inferCNV. The window size of 101 genes is smoothed to calculate the CNV scores. The relative expression values of analyzed genes were bounded to −1 to 1.

**Cell-cell interaction analysis**

The human ligand and receptor pairs list were downloaded from FANTOM (<https://fantom.gsc.riken.jp/5/suppl/Ramilowski_et_al_2015/data/PairsLigRec.txt>). Then the cell-cell interaction analysis was based on the expression of these receptors and ligands. We only selected receptors and ligands that were expressed in > 10% of the cells in the analyzed cluster. The significance of the interaction was then evaluated using a random permutation of the samples. Then we screened the differentially expressed genes between cases and controls (|log2FC| ≥ 0.585 and FDR ≤ 0.05) in each cell type. If the ligand and receptor pair were up-regulated at the same time, it was considered that the cell type interaction (Gain) was obtained, and the ligand and receptor pair were down-regulated at the same time (Loss), and the cell type interaction was considered to be lost.

**Statistical analysis**

Statistics analysis were performed with GraphPad Prism v8.0. Significant difference between independent two groups were analyzed via student’s two-tailed t-test. The results of were expressed as the mean ± s.d. from at least three independent experiments. And the variance was similar between the groups that were being statistically compared. Gene expression data of MF were retrieved from GSE12902 in GEO. 22 cases of MF samples were recruited and divided into two groups according to low and high expression of genes for survival analysis (Table S9). Survival curves were plotted using the Kaplan-Meier method and compared by the log-rank test. *P* < 0.05 was considered statistically significant.

**Supplemental References**

1. Kohnken R, Fabbro S, Hastings J, Porcu P, Mishra A. Sézary syndrome: clinical and biological aspects. Curr Hematol Malig Rep. 2016;11:468-79.

2. Satija R, Farrell JA, Gennert D, Schier AF, Regev A. Spatial reconstruction of single-cell gene expression data. Nat Biotechnol. 2015;33:495-502.

3. Wolock SL, Lopez R, Klein AM. Scrublet: Computational identification of cell doublets in single-cell transcriptomic data. Cell Syst. 2019;8:281-91 e9.

4. Bergen V, Lange M, Peidli S, Wolf FA, Theis FJ. Generalizing RNA velocity to transient cell states through dynamical modeling. Nat Biotechnol. 2020;38:1408-14.

5. La Manno G, Soldatov R, Zeisel A, Braun E, Hochgerner H, Petukhov V, et al. RNA velocity of single cells. Nature. 2018;560:494-8.

6. T. Tickle, I. Tirosh, C. Georgescu, M. Brown, B. Haas. inferCNV of the Trinity CTAT Project. Klarman Cell Observatory, Broad Institute of MIT and Harvard, Cambridge, MA, USA. https://github.com/broadinstitute/inferCNV (2019).
