# Supplementary material for: Single-cell analyses reveal novel molecular signatures and pathogenesis in cutaneous T cell lymphoma
Source: Cell Death Dis. 2022 Nov 18;13(11):970. doi: 10.1038/s41419-022-05323-5 (PMC9674677; doi:10.1038/s41419-022-05323-5)
Supplement: Supplementary file 2 — Supplementary figures 1-9 and figure legends [file 41419_2022_5323_MOESM2_ESM.pdf]

Supplementary figures and figure legends

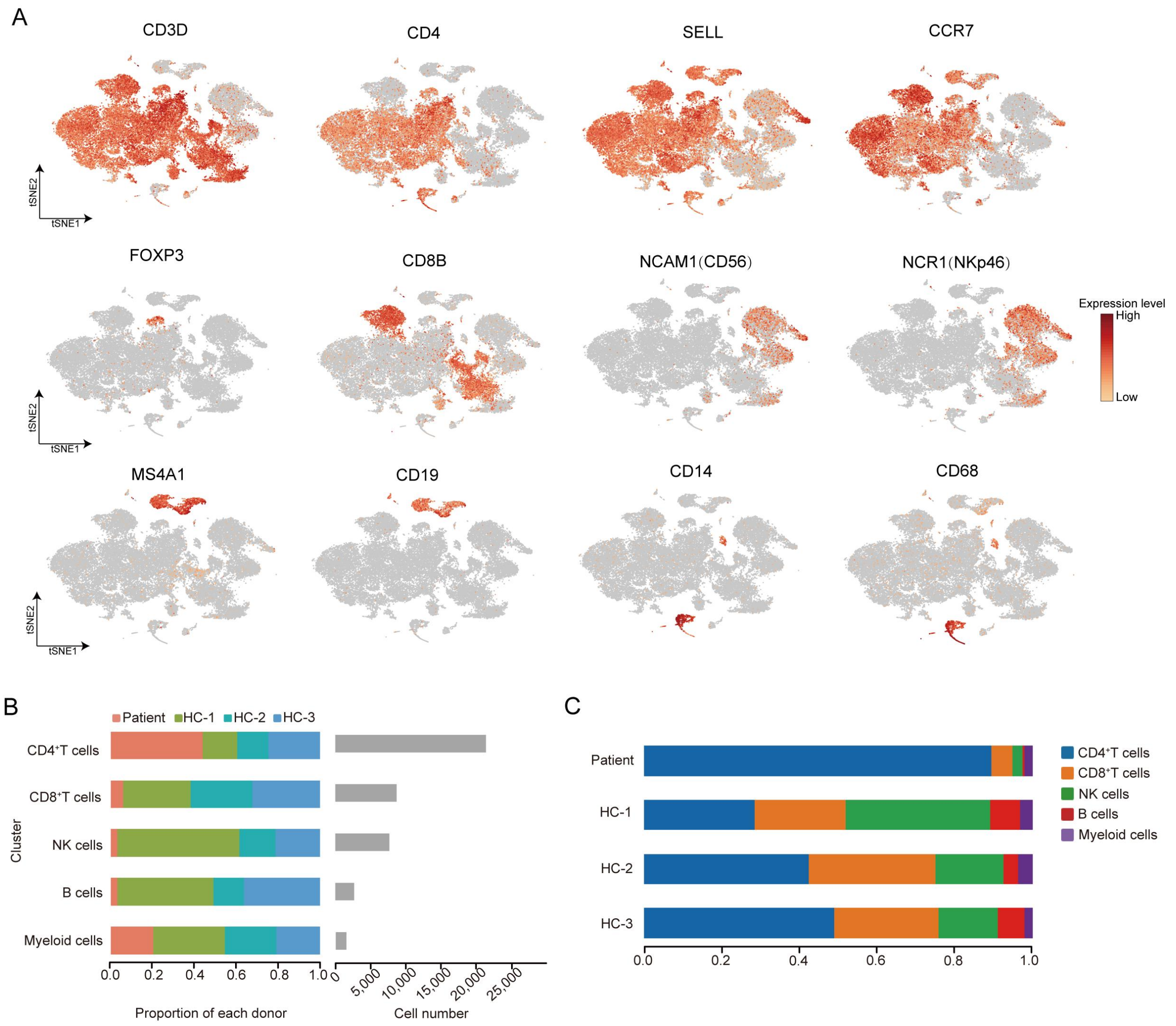

Supplementary Fig. S1 Clustering of PBMCs from one SS patient and three HCs. (A) t-SNE plots showing the expression of selected marker genes in major immune cell types of PBMCs. **(B)** The donor composition and cell numbers of five main cell types. **(C)** Cell percentages of five main clusters in each donor.

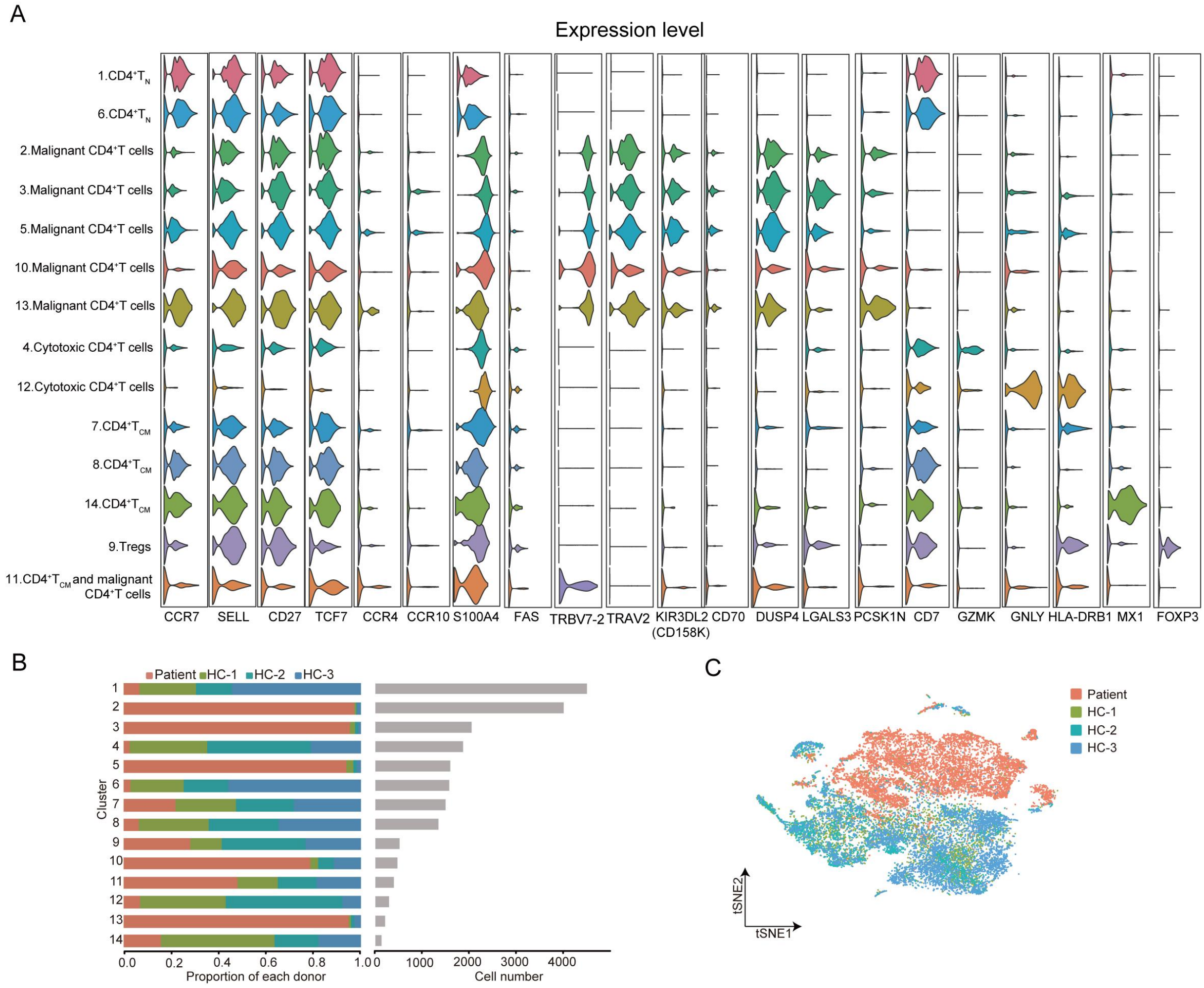

Supplementary Fig. S2 Sub-clustering of PBMC CD4$^+$ T cells from the SS patient and three HCs. (A) The expression of discriminative marker genes used for the identification of PBMC CD4$^+$ T cell subtypes indicated by violin plots. **(B)** Donor composition and cell numbers for each cluster obtained in the sub-clustering of PBMC CD4$^+$ T cells. **(C)** t-SNE plot for PBMC CD4$^+$ T cells split by the SS patient and three HCs.

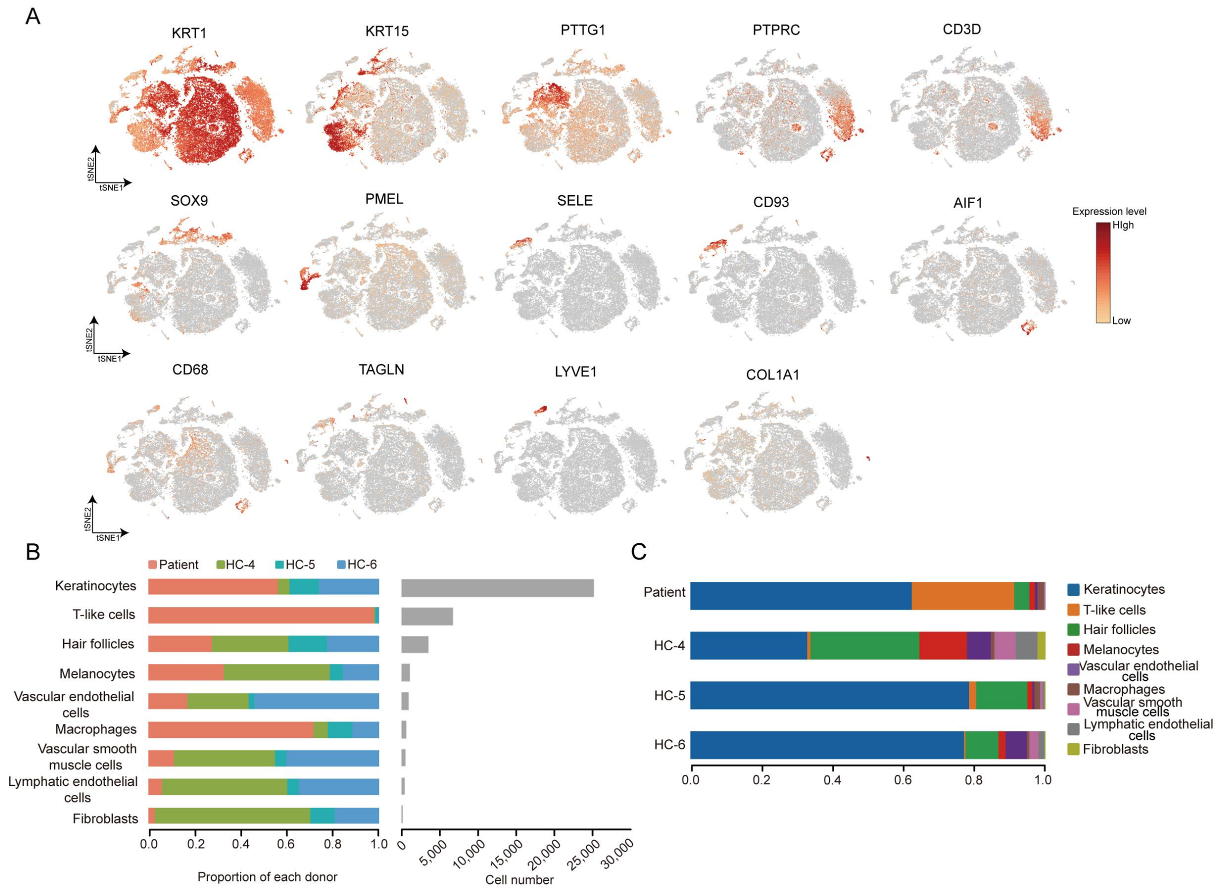

Supplementary Fig. S3 Clustering of skin cells from one SS patient and three HCs. (A) t-SNE plots showing the expression of major discriminative marker genes for cell types identification of skin cells. **(B)** The donor composition and cell numbers of nine main cell types. **(C)** Cell percentages of nine main clusters in each donor.

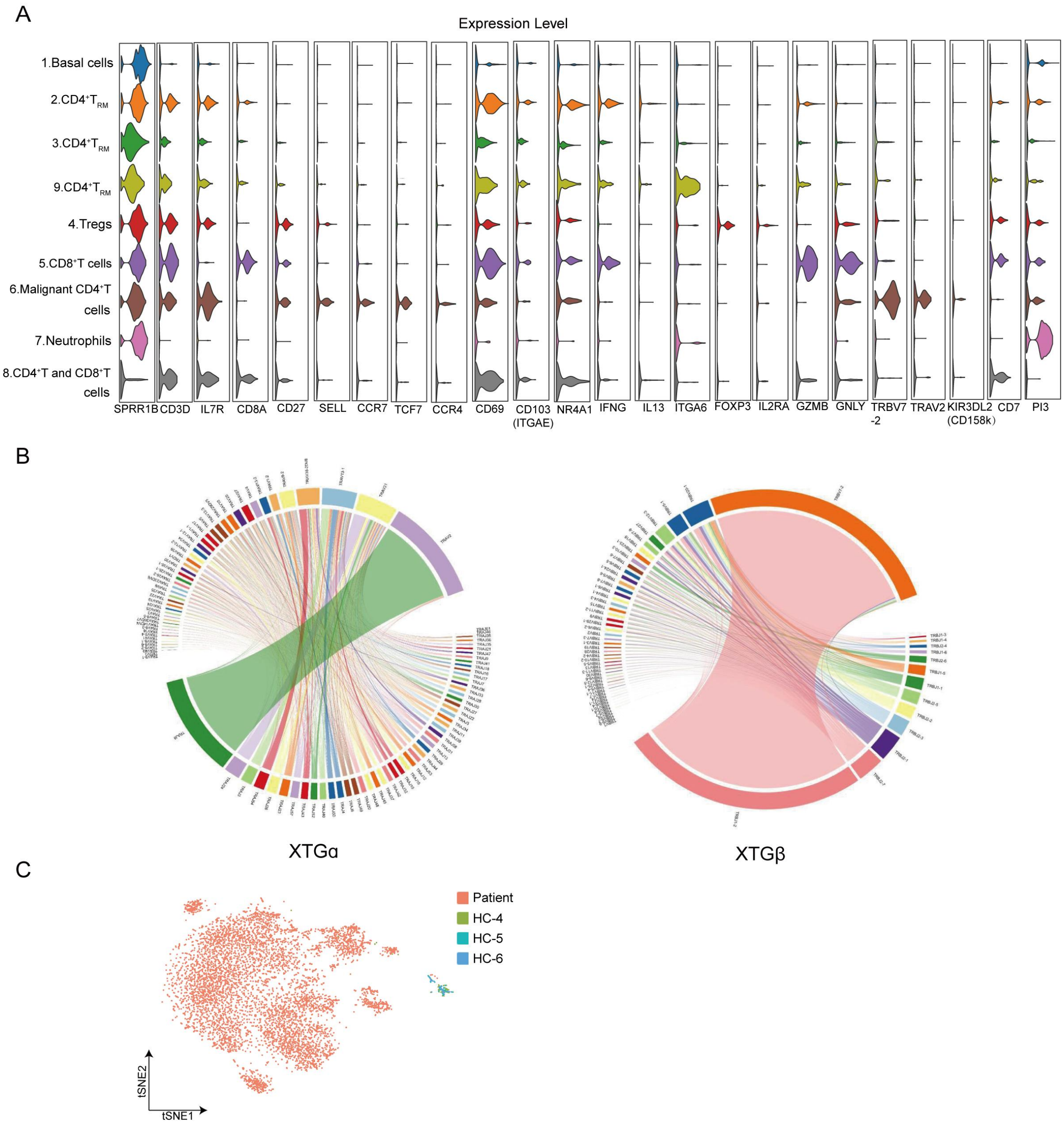

Supplementary Fig. S4 Sub-clustering of skin T-like cells from the SS patient and three HCs. (A) The expression of discriminative marker genes used for the identification of skin T-like cell subtypes indicated by violin plots. **(B)** V-J junction Circos images showing TRBV7-2 and TRAV2 had higher frequency in skin sample of the SS patient. **(C)** t-SNE plot for skin T-like cells split by the SS patient and three HCs.

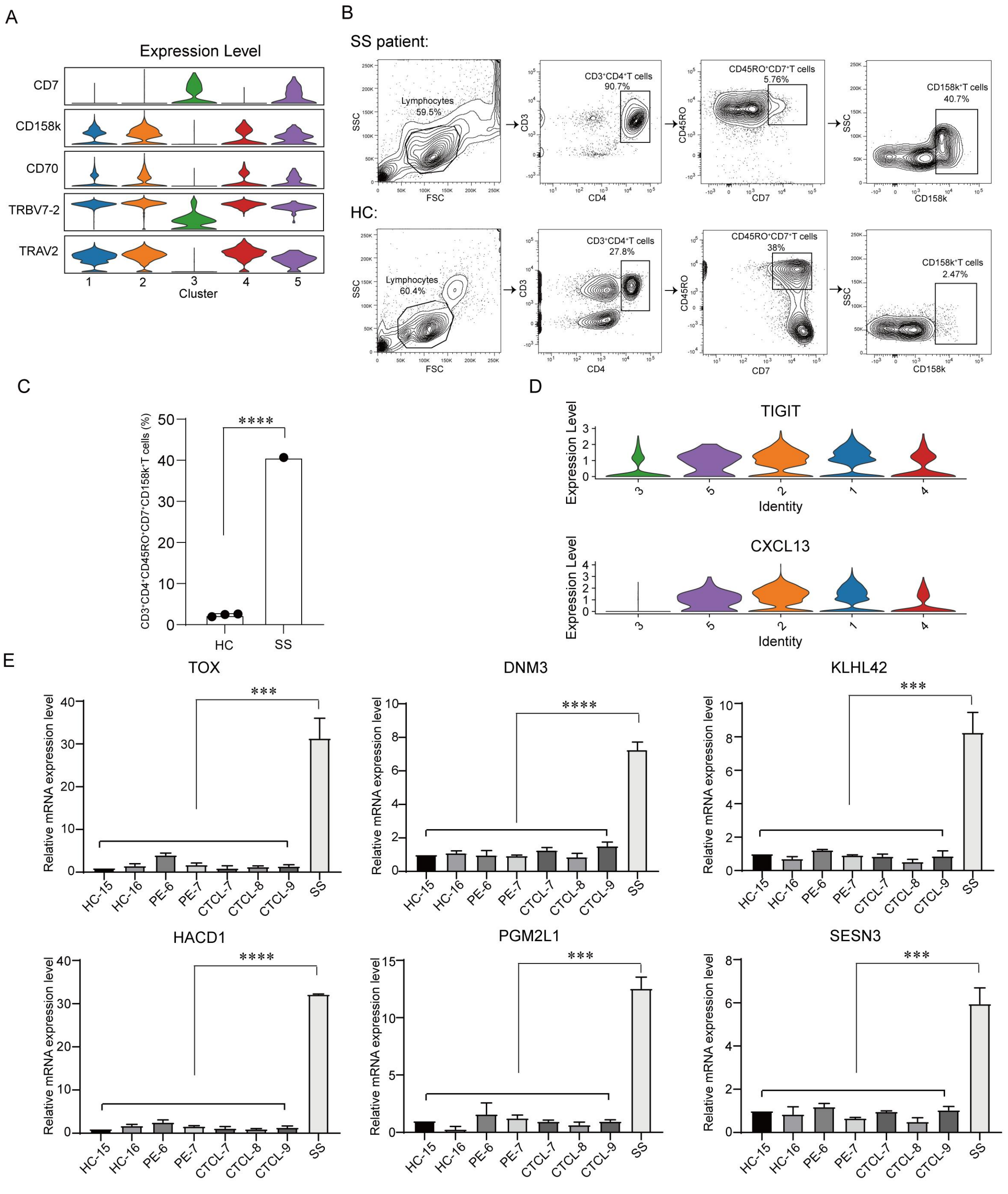

Supplementary Fig. S5 A series of specific marker genes were correlated with the malignant degree of circulating CD4$^+$ T cells. (A) Violin plots showing the expressions of CD7, CD158k, CD70, TRBV7-2, and TRAV2 in each cluster. **(B-C)** Intermediated CD4$^+$ T cell subset was validated in the SS patient and three HCs by FC. **(D)** Violin plots showing the expression of *TIGIT* and *CXCL13* in each cluster from PBMC CD4$^+$ T cells of the SS patient. **(E)** The mRNA expression of *TOX*, *DNM3*, *KLHL42*, *HACD1*, *PGM2L1*, and *SESN3* in

circulating $CD4^+$ T cells in two HCs, two PE patients, three advanced-stage MF patients, and one SS patient was measured by qRT-PCR. Data are presented as mean ± s.d. ***$P < 0.001$, ****$P < 0.0001$.

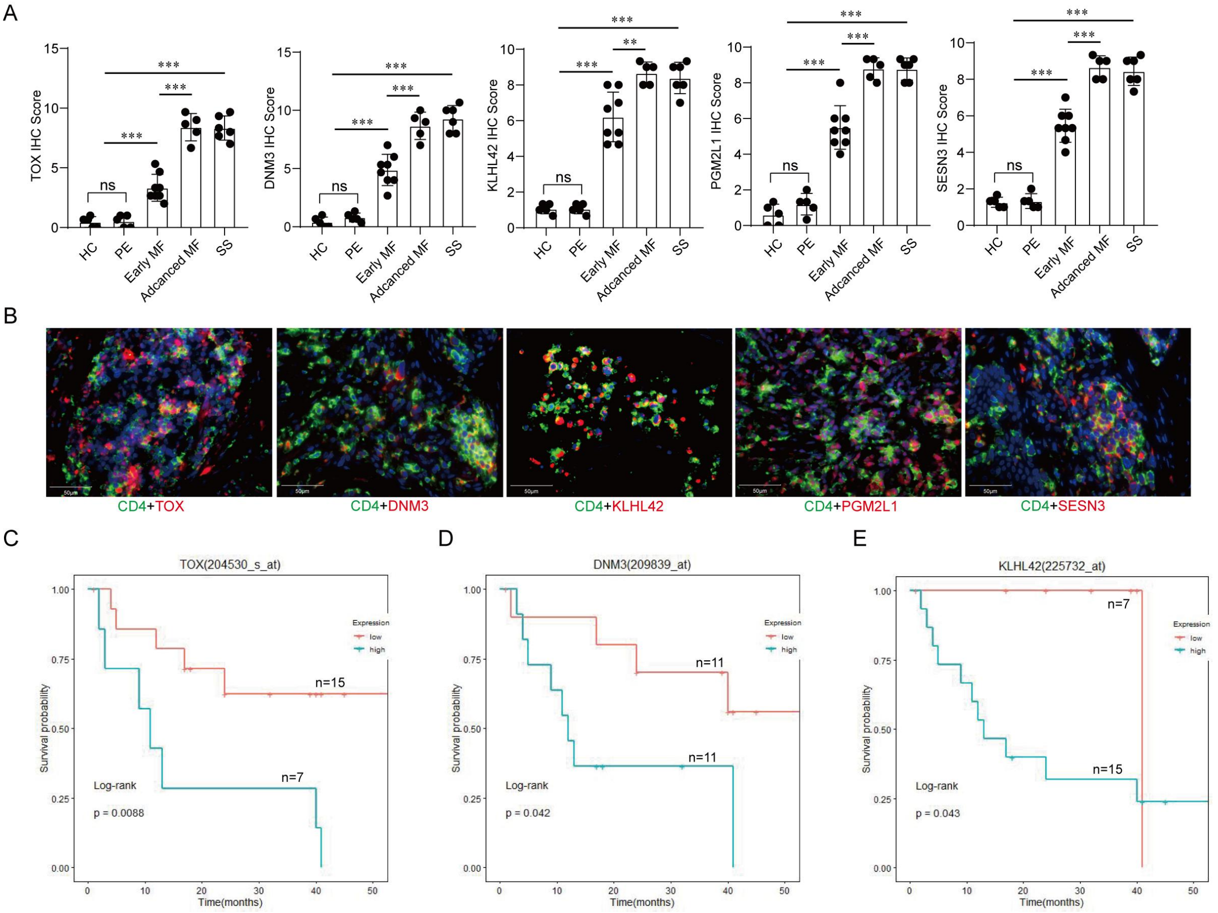

Supplementary Fig. S6 A series of specific marker genes were correlated with the disease progression of MF/SS. (A) Immunohistochemical statistical analysis of IHC scores of *TOX*, *DNM3*, *KLHL42*, *HACD1*, *PGM2L1*, and *SESN3* in skin biopsies of HCs (n=5), PE patients (n=5), early-stage MF patients (n=8), advanced-stage MF patients (n=5), and SS patients (n=6). Data are presented as mean ± s.d. ns, no significant, $**P < 0.01$, $***P < 0.001$. **(B)** Representative skin examples from SS patients tested of double color immunofluorescence staining for CD4/SESN3, CD4/KLHL42, CD4/PGM2L1, CD4/DNM3, and CD4/TOX, as indicated, at 200×. DAPI stains nuclei. **(C-E)** Kaplan-Meier plots predicting suvival rate in MF based on the tumor-associated genes (*TOX*, *DNM3*, and *KLHL42*).

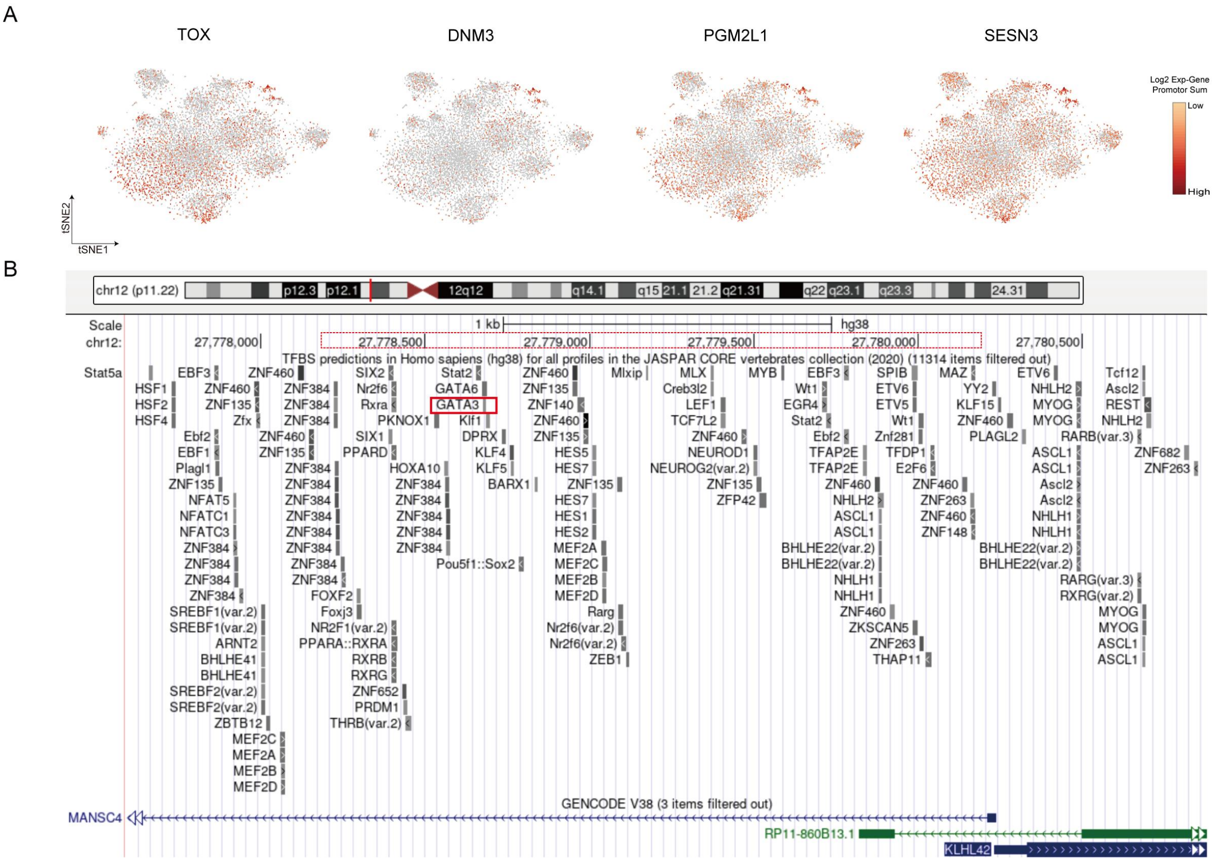

Supplementary Fig. S7 The expression of tumor-associated genes (*TOX*, *DNM3*, *PGM2L1*, and *SESN3*) wasn't significantly influenced by epigenetic modification, and *GATA3* could bind to the promoter region of *KLHL42*. (A) t-SNE projection colored by log2 Exp-gene promoter sum demonstrating the chromatin accessibility of *TOX*, *DNM3*, *PGM2L1*, and *SESN3*. **(B)** TF motif analysis of promoter region (chr12: 27778233-27780233) of *KLHL42* by combining JASPAR2020 TFBS hg38 database and UCSC database (Track score: 481, $P \leq 10^{-4}$).

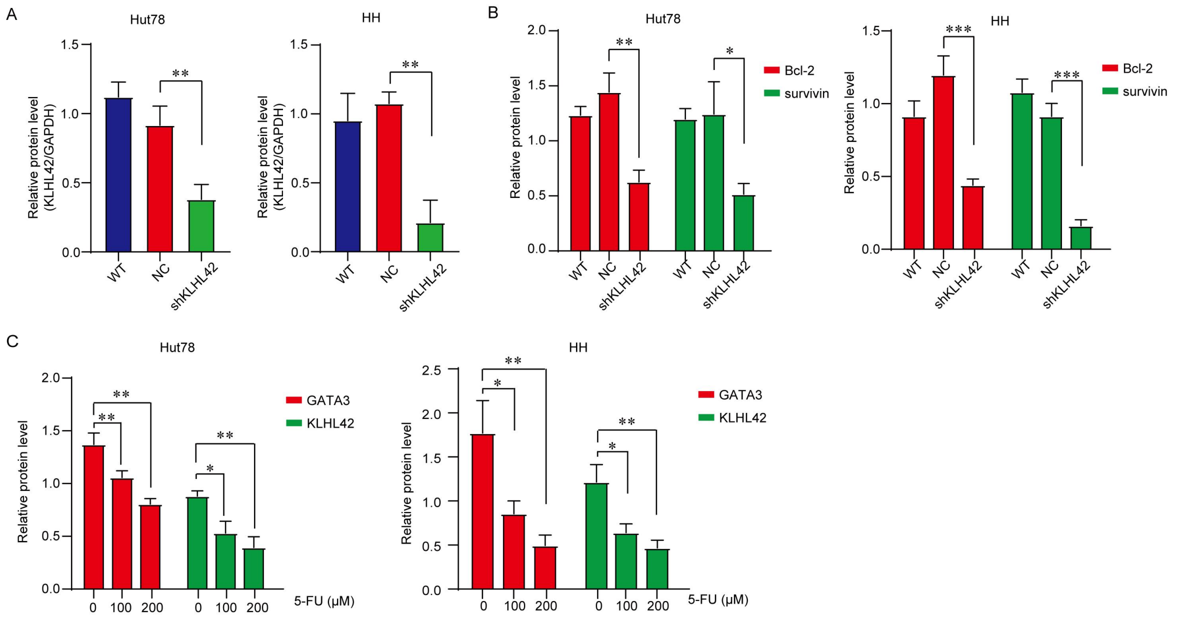

Supplementary Fig. S8 *KLHL42* knockdown could promoted Hut78 and HH cell apoptosis, and 5-FU could repress the expression of *GATA3* and *KLHL42*. (A) Knockdown of *KLHL42* was confirmed in Hut78 and HH cells by western blotting. **(B)** *KLHL42* knockdown decreased the expression of Bcl-2 and survivin. **(C)** 5-FU repressed the *GATA3* and *KLHL42* expression in a dose-dependent manner. The protein levels of both *GATA3* and *KLHL42* were measured by western blotting. Data are presented as mean ± s.d. *$P < 0.05$, **$P < 0.01$, ***$P < 0.001$.

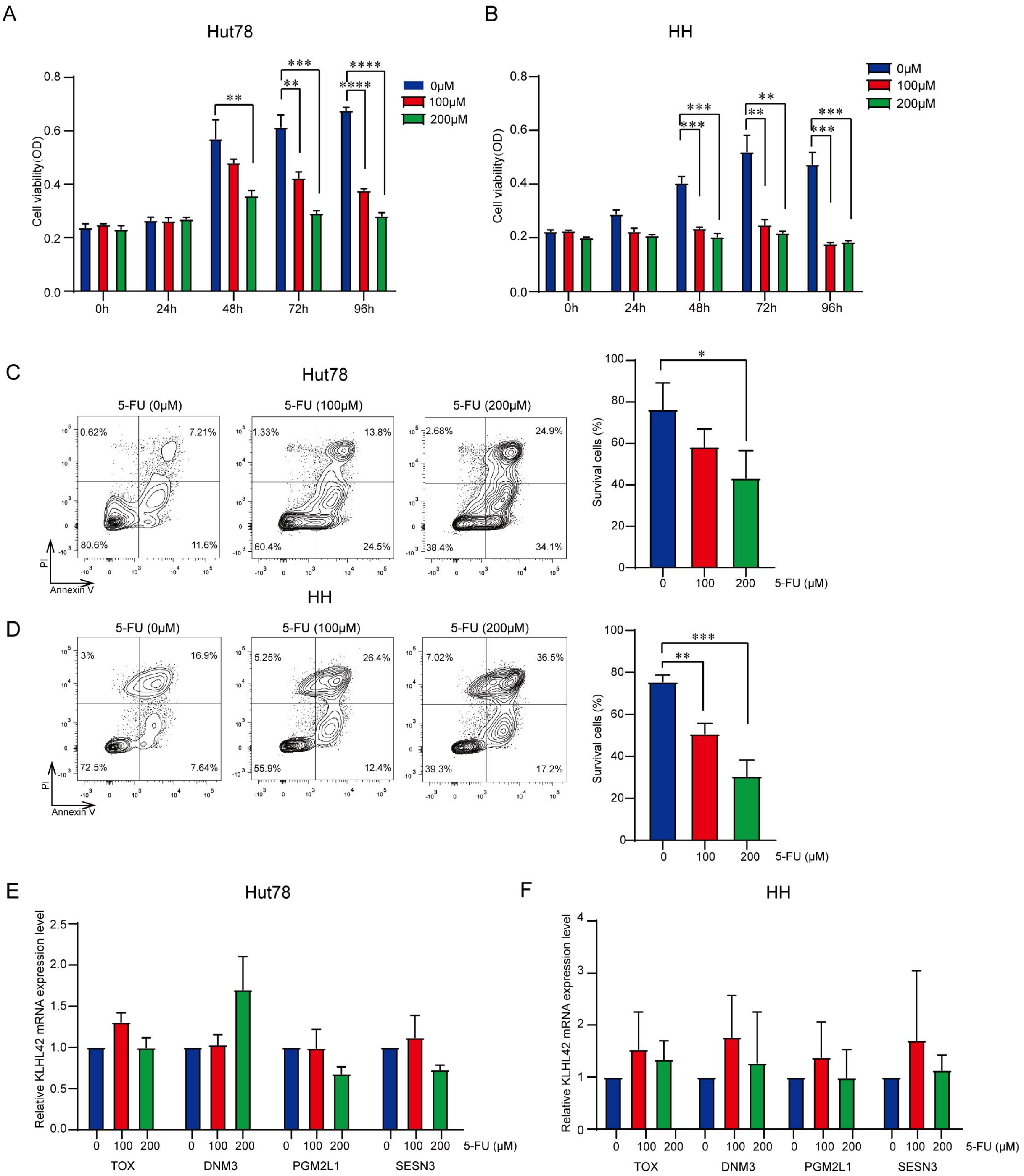

Supplementary Fig. S9 5-FU could inhibit Hut78 and HH cell viability and induced its apoptosis *in vitro*, and 5-FU couldn't repress the expression of other tumor-associated genes. (A-B) CCK-8 proliferation assays showing that 5-FU repressed Hut78 and HH cell viability in a dose-dependent manner. **(C-D)** Cell viability was further examined by flow cytometry in Hut78 and HH cells after exposure (48 h) to 5-FU at the concentrations shown (0 μM, 100 μM, 200 μM). **(E-F)** The mRNA expression of tumor-associated genes (*TOX*, *DNM3*, *PGM2L1*, and *SESN3*) in Hut78 and HH cells after the treatment of 5-FU was detected. Data

are presented as mean ± s.d. *$P < 0.05$,**$P < 0.01$, ***$P < 0.001$, ****$P < 0.0001$.
