# Supplementary material for: Single-cell analyses reveal novel molecular signatures and pathogenesis in cutaneous T cell lymphoma
Source: Cell Death Dis. 2022 Nov 18;13(11):970. doi: 10.1038/s41419-022-05323-5 (PMC9674677; doi:10.1038/s41419-022-05323-5)
Supplement: Supplementary file 3 — Supplementary tables 1-9 [file 41419_2022_5323_MOESM3_ESM.doc]

**Supplementary tables**

**Supplementary Table S1.**

1. **Cell numbers of each donor in each cluster of PBMCs from one SS patient and three HCs for scRNA-seq.**

| **Cell type** | **Cluster** | **Cell numbers of each donor** | | | | **Total of each cluster** |
| --- | --- | --- | --- | --- | --- | --- |
|  |  | **HC-1** | **HC-2** | **HC-3** | **SS** |  |
| CD4^+^ T cells | Cluster 1 | 3302 | 3092 | 5104 | 9198 | 20696 |
| CD8^+^ T cells | Cluster 2 | 2770 | 2437 | 2787 | 550 | 8544 |
| NK cells | Cluster 3 | 4407 | 1339 | 1627 | 275 | 7648 |
| B cells | Cluster 4 | 948 | 299 | 755 | 74 | 2076 |
| Myeloid cells | Cluster 5 | 466 | 335 | 286 | 282 | 1369 |
| Total of each donor | | 11893 | 7502 | 10559 | 10379 | 40333 |

1. **Cell numbers of each donor in each cluster of PBMC CD4^+^ T cells from the SS patient and three HCs for scRNA-seq.**

| **Cell type** | **Cluster** | **Cell numbers of each donor** | | | | **Total of each cluster** |
| --- | --- | --- | --- | --- | --- | --- |
|  |  | **HC-1** | **HC-2** | **HC-3** | **SS** |  |
| CD4^+^ T_N_ | Cluster 1 | 1089 | 687 | 2479 | 304 | 4559 |
| Malignant CD4^+^ T cells | Cluster 2 | 37 | 11 | 72 | 3941 | 4061 |
| Malignant CD4^+^ T cells | Cluster 3 | 45 | 28 | 25 | 1975 | 2073 |
| Cytotoxic CD4^+^ T cells | Cluster 4 | 614 | 824 | 400 | 50 | 1888 |
| Malignant CD4^+^ T cells | Cluster 5 | 48 | 23 | 30 | 1514 | 1615 |
| CD4^+^ T_N_ | Cluster 6 | 357 | 299 | 891 | 45 | 1592 |
| CD4^+^ T_CM_ | Cluster 7 | 388 | 370 | 429 | 329 | 1516 |
| CD4^+^ T_CM_ | Cluster 8 | 399 | 399 | 473 | 88 | 1359 |
| Tregs | Cluster 9 | 69 | 183 | 122 | 146 | 520 |
| Malignant CD4^+^ T cells | Cluster 10 | 16 | 31 | 55 | 375 | 477 |
| CD4^+^ T_CM_ and malignant CD4^+^ T cells | Cluster 11 | 68 | 65 | 75 | 191 | 399 |
| Cytotoxic CD4^+^ T cells | Cluster 12 | 106 | 144 | 23 | 20 | 293 |
| Malignant CD4^+^ T cells | Cluster 13 | 2 | 3 | 6 | 199 | 210 |
| CD4^+^ T_CM_ | Cluster 14 | 64 | 25 | 24 | 21 | 134 |
| Total of each donor | | 3302 | 3092 | 5104 | 9198 | 20696 |

**Abbreviations:** CD4^+^ T_N_, Naive CD4^+^ T cells; CD4^+^ T_CM_, Central memory CD4^+^ T cells.

**Supplementary Table S2.**

1. **Cell numbers of each donor in each cluster of skin cells from one SS patient and three HCs for scRNA-seq.**

| **Cell type** | **Cluster** | **Cell numbers of each donor** | | | | **Total of each cluster** |
| --- | --- | --- | --- | --- | --- | --- |
|  |  | **HC-4** | **HC-5** | **HC-6** | **SS** |  |
| Keratinocytes | Cluster 1 | 1315 | 3392 | 7007 | 15047 | 26761 |
| T-like cells | Cluster 2 | 35 | 87 | 45 | 6975 | 7142 |
| Hair follicles | Cluster 3 | 1231 | 619 | 839 | 1027 | 3716 |
| Melanocytes | Cluster 4 | 534 | 66 | 184 | 383 | 1167 |
| Vascular endothelial cells | Cluster 5 | 268 | 24 | 546 | 172 | 1010 |
| Macrophages | Cluster 6 | 40 | 68 | 75 | 459 | 642 |
| Vascular smooth muscle cells | Cluster 7 | 242 | 27 | 223 | 62 | 554 |
| Lymphatic endothelial cells | Cluster 8 | 245 | 23 | 158 | 28 | 454 |
| Fibroblasts | Cluster 9 | 90 | 14 | 26 | 4 | 134 |
| Total of each donor | | 4000 | 4320 | 9103 | 24157 | 41580 |

1. **Cell numbers of each donor in each cluster of skin T-like cells from the SS patient and three HCs for scRNA-seq.**

| **Cell type** | **Cluster** | **Cell numbers of each donor** | | | | **Total of each cluster** |
| --- | --- | --- | --- | --- | --- | --- |
|  |  | **HC-4** | **HC-5** | **HC-6** | **SS** |  |
| Basal cells | Cluster 1 | 0 | 1 | 0 | 3739 | 3740 |
| CD4^+^ T_RM_ | Cluster 2 | 0 | 0 | 0 | 1469 | 1469 |
| CD4^+^ T_RM_ | Cluster 3 | 0 | 5 | 0 | 596 | 601 |
| Tregs | Cluster 4 | 0 | 2 | 0 | 390 | 392 |
| CD8^+^ T cells | Cluster 5 | 0 | 0 | 0 | 242 | 242 |
| Malignant CD4^+^ T cells | Cluster 6 | 0 | 0 | 0 | 237 | 237 |
| Neutrophils | Cluster 7 | 0 | 0 | 0 | 171 | 171 |
| CD4^+^ T and CD8^+^ T cells | Cluster 8 | 35 | 77 | 45 | 7 | 164 |
| CD4^+^ T_RM_ | Cluster 9 | 0 | 2 | 0 | 124 | 126 |
| Total of each donor | | 35 | 87 | 45 | 6975 | 7142 |

**Abbreviations:** CD4^+^ T_RM_, Resident memory CD4^+^ T cells.

**Supplementary Table S3. Top DEGs (excluding ribosomal associated genes) of malignant vs benign CD4^+^ T cells of PBMCs from the SS patient and three HCs.**

(A) Upregulated genes (Log2FC > 0 and Top50 of Log2FC, pct.1 ≥ 0.25, pct.2 ≤ 0.25)

| Symbol | pct.1 | pct.2 | malignant_Mean_UMI_Counts | normal_Mean_UMI_Counts | Log2_Fold_Change | Pvalue | Adjust Pvalue |
| --- | --- | --- | --- | --- | --- | --- | --- |
| TRBV7-2 | 0.93 | 0.128 | 43.54315669 | 3.498520509 | 3.307681298 | 0 | 0 |
| TRAV2 | 0.827 | 0.072 | 4.616404083 | 0.340150429 | 2.067251787 | 0 | 0 |
| CXCL13 | 0.638 | 0.026 | 3.319296356 | 0.099020347 | 1.97457821 | 0 | 0 |
| LGALS1 | 0.869 | 0.237 | 8.599683469 | 1.553213513 | 1.910672648 | 0 | 0 |
| DUSP4 | 0.748 | 0.059 | 3.325441581 | 0.18562355 | 1.867201414 | 0 | 0 |
| TRBV23-1 | 0.657 | 0.034 | 2.395272482 | 0.133314917 | 1.582978558 | 0 | 0 |
| MYOM2 | 0.669 | 0.078 | 2.458252554 | 0.208522786 | 1.516798558 | 0 | 0 |
| CRIP2 | 0.707 | 0.225 | 3.31036902 | 0.70304719 | 1.339692975 | 0 | 0 |
| TIGIT | 0.664 | 0.126 | 2.334012341 | 0.431309341 | 1.219923937 | 0 | 0 |
| KIR3DL2 | 0.548 | 0.028 | 1.406253079 | 0.065045137 | 1.175873814 | 0 | 0 |
| LMNA | 0.597 | 0.143 | 2.111144991 | 0.501150225 | 1.051377273 | 0 | 0 |
| TOX | 0.522 | 0.073 | 1.392274405 | 0.171249544 | 1.030334397 | 0 | 0 |
| RTKN2 | 0.495 | 0.054 | 1.363912719 | 0.159205086 | 1.028040939 | 0 | 0 |
| DNM3 | 0.457 | 0.023 | 1.125938258 | 0.04858277 | 1.019658954 | 0 | 0 |
| PGM2L1 | 0.496 | 0.088 | 1.323585051 | 0.195544667 | 0.958684421 | 0 | 0 |
| JPT1 | 0.633 | 0.242 | 2.116531058 | 0.605327813 | 0.95707316 | 0 | 0 |
| PTHLH | 0.393 | 0.014 | 0.963711969 | 0.026954041 | 0.935211717 | 0 | 0 |
| TUBA1C | 0.579 | 0.175 | 1.697762315 | 0.42278374 | 0.923046853 | 0 | 0 |
| PLS3 | 0.409 | 0.018 | 0.948246705 | 0.035009442 | 0.912532448 | 0 | 0 |
| MFSD10 | 0.66 | 0.248 | 2.001914454 | 0.611001251 | 0.897925251 | 0 | 0 |
| SLF1 | 0.465 | 0.095 | 1.276192515 | 0.226778974 | 0.891747238 | 0 | 0 |
| KRT7 | 0.408 | 0.019 | 0.919842202 | 0.035492152 | 0.890671117 | 0 | 0 |
| LGALS3 | 0.511 | 0.099 | 1.498500417 | 0.358618426 | 0.878922133 | 0 | 0 |
| HACD1 | 0.406 | 0.03 | 0.952757535 | 0.062759046 | 0.877698288 | 0 | 0 |
| STAM | 0.517 | 0.127 | 1.372121294 | 0.317928149 | 0.847906062 | 0 | 0 |
| KLHL42 | 0.418 | 0.054 | 1.002022737 | 0.115796464 | 0.843384475 | 0 | 0 |
| PCSK1N | 0.381 | 0.078 | 1.139381441 | 0.198147254 | 0.836388502 | 0 | 0 |
| SESN3 | 0.546 | 0.194 | 1.660552919 | 0.504213883 | 0.822716382 | 0 | 0 |

(B) Downregulated genes (Log2FC < 0 and Top50 of Log2FC, pct.1 ≤ 0.25, pct.2 ≥ 0.25)

| Symbol | pct.1 | pct.2 | malignant_Mean_UMI_Counts | normal_Mean_UMI_Counts | Log2_Fold_Change | Pvalue | Adjust Pvalue |
| --- | --- | --- | --- | --- | --- | --- | --- |
| SH3YL1 | 0.046 | 0.317 | 0.113738103 | 0.883521313 | -0.758022338 | 0 | 0 |
| ACTN1 | 0.015 | 0.28 | 0.057348891 | 0.810515667 | -0.775949162 | 0 | 0 |
| KLF3 | 0.095 | 0.407 | 0.280445313 | 1.248636715 | -0.812404963 | 0 | 0 |
| PCED1B | 0.052 | 0.334 | 0.13813637 | 1.002776081 | -0.8153277 | 0 | 0 |
| PDCD4 | 0.205 | 0.486 | 0.435741024 | 1.545760658 | -0.826301247 | 0 | 0 |
| AC243960.1 | 0.21 | 0.495 | 0.4531728 | 1.6306781 | -0.856228458 | 0 | 0 |
| PLAC8 | 0.021 | 0.335 | 0.092793216 | 0.984810864 | -0.860981104 | 0 | 0 |
| ITGA4 | 0.037 | 0.337 | 0.169809646 | 1.169128177 | -0.890841516 | 0 | 0 |
| C1orf162 | 0.071 | 0.364 | 0.156627755 | 1.152651758 | -0.89619032 | 0 | 0 |
| ABLIM1 | 0.12 | 0.426 | 0.313460139 | 1.478989309 | -0.916379631 | 0 | 0 |
| GSTP1 | 0.043 | 0.396 | 0.135419759 | 1.144354915 | -0.917317954 | 0 | 0 |
| IFITM3 | 0.117 | 0.464 | 0.27400519 | 1.596667943 | -1.027290382 | 0 | 0 |
| GZMM | 0.03 | 0.425 | 0.122032029 | 1.308345825 | -1.040745518 | 0 | 0 |
| SATB1 | 0.033 | 0.422 | 0.12449583 | 1.371019382 | -1.076229145 | 0 | 0 |
| CD6 | 0.236 | 0.644 | 0.592435205 | 2.443283635 | -1.112550354 | 0 | 0 |
| LINC00861 | 0.051 | 0.526 | 0.215222971 | 1.894571209 | -1.252128602 | 0 | 0 |
| MGAT4A | 0.038 | 0.556 | 0.194291393 | 1.92611163 | -1.292829928 | 0 | 0 |
| CD7 | 0.046 | 0.694 | 0.26045137 | 3.713174102 | -1.902758517 | 0 | 0 |
| MTRNR2L12 | 0.073 | 0.647 | 1.338320972 | 9.515765326 | -2.16900897 | 0 | 0 |

**Abbreviations:** DEGs, Differentially expressed genes.

**Supplementary Table S4. DEGs (excluding ribosomal associated genes) of malignant CD4^+^T cells vs other benign immune cells of skin from the SS patient and three HCs.**

1. Upregulated genes (Log2FC ≥ 0.5, pct.1 ≥ 0.25, pct.2 ≤ 0.25)

| gene | pct.1 | pct.2 | malignant_Mean_UMI_Counts | Other_Mean_UMI_Counts | Log2_Fold_Change | Pvalue | Adjust Pvalue |
| --- | --- | --- | --- | --- | --- | --- | --- |
| CXCL13 | 0.743 | 0.083 | 31.15926311 | 0.704428697 | 4.237874197 | 2.3302E-148 | 4.3852E-144 |
| TRBV7-2 | 0.821 | 0.08 | 18.51376739 | 0.85383178 | 3.395910094 | 8.7582E-166 | 1.6482E-161 |
| TRBV23-1 | 0.56 | 0.018 | 3.632998625 | 0.044642889 | 2.148936412 | 1.3402E-129 | 2.522E-125 |
| LINC01480 | 0.358 | 0.011 | 3.428272575 | 0.039786563 | 2.090456611 | 3.58013E-76 | 6.73745E-72 |
| TRAV2 | 0.482 | 0.025 | 3.187923268 | 0.104028405 | 1.923457718 | 2.85232E-92 | 5.36777E-88 |
| ITGB1 | 0.661 | 0.247 | 5.907166792 | 1.099525239 | 1.71803093 | 1.17618E-59 | 2.21345E-55 |
| SESN3 | 0.427 | 0.079 | 3.104908873 | 0.289359363 | 1.670695775 | 1.85251E-50 | 3.48624E-46 |
| AHI1 | 0.427 | 0.093 | 3.18031066 | 0.366604112 | 1.613014787 | 3.00465E-45 | 5.65445E-41 |
| IKZF2 | 0.294 | 0.059 | 2.234607886 | 0.20142971 | 1.428838588 | 4.27557E-34 | 8.04619E-30 |
| SCG2 | 0.303 | 0.011 | 1.757283013 | 0.036415441 | 1.411644941 | 1.60741E-61 | 3.02499E-57 |
| KLHL42 | 0.33 | 0.05 | 2.057163043 | 0.170096453 | 1.385566037 | 8.31033E-40 | 1.56392E-35 |
| PGM2L1 | 0.381 | 0.039 | 1.911107408 | 0.130161064 | 1.365039679 | 5.94194E-56 | 1.11821E-51 |
| ITK | 0.422 | 0.142 | 3.031334683 | 0.603836551 | 1.329730439 | 5.79705E-29 | 1.09095E-24 |
| CPM | 0.321 | 0.052 | 1.944352997 | 0.178008476 | 1.321600725 | 5.00572E-38 | 9.42026E-34 |
| RBPJ | 0.505 | 0.213 | 3.561215879 | 0.849905772 | 1.301966667 | 4.7792E-33 | 8.99397E-29 |
| TCF7 | 0.367 | 0.036 | 1.739402689 | 0.120272522 | 1.290011626 | 4.62853E-56 | 8.71044E-52 |
| SELL | 0.312 | 0.037 | 1.759901042 | 0.157199334 | 1.253979141 | 4.38123E-39 | 8.24503E-35 |
| HDAC9 | 0.266 | 0.015 | 1.489029547 | 0.044748978 | 1.252427009 | 5.82977E-49 | 1.0971E-44 |
| CCR7 | 0.294 | 0.032 | 1.646480191 | 0.117620388 | 1.243644611 | 1.52472E-38 | 2.86938E-34 |
| TSHZ2 | 0.45 | 0.166 | 2.910424727 | 0.68767804 | 1.212285607 | 1.89291E-29 | 3.56227E-25 |
| TIGIT | 0.404 | 0.105 | 2.376890422 | 0.463457789 | 1.206314232 | 4.13396E-31 | 7.7797E-27 |
| INPP4B | 0.372 | 0.069 | 1.905360901 | 0.260777203 | 1.204404031 | 7.18857E-36 | 1.35282E-31 |
| SFXN1 | 0.445 | 0.176 | 2.801402587 | 0.65025699 | 1.203841113 | 9.77167E-31 | 1.83893E-26 |
| GIMAP7 | 0.344 | 0.074 | 2.008462989 | 0.315844515 | 1.193037583 | 1.18709E-29 | 2.23399E-25 |
| PDE7B | 0.261 | 0.038 | 1.647472546 | 0.159650717 | 1.190925387 | 4.31359E-28 | 8.11774E-24 |
| DNM3 | 0.266 | 0.013 | 1.285203593 | 0.038082274 | 1.138401913 | 1.20945E-49 | 2.27607E-45 |
| LIMD2 | 0.578 | 0.239 | 3.744180617 | 1.175933272 | 1.124524621 | 2.1256E-30 | 4.00018E-26 |
| GPR174 | 0.431 | 0.151 | 2.629558424 | 0.690370782 | 1.102454304 | 3.12716E-24 | 5.88501E-20 |
| HACD1 | 0.257 | 0.018 | 1.196911828 | 0.065439075 | 1.044028871 | 1.64246E-41 | 3.09095E-37 |
| TOX | 0.317 | 0.064 | 1.557703913 | 0.245520878 | 1.038100058 | 2.15213E-27 | 4.0501E-23 |
| TRAF1 | 0.349 | 0.091 | 1.633107014 | 0.352826451 | 0.960789383 | 5.62683E-27 | 1.05891E-22 |
| RIPOR2 | 0.294 | 0.083 | 1.489750764 | 0.322229029 | 0.913029234 | 1.16883E-18 | 2.19963E-14 |
| TRAF3IP3 | 0.335 | 0.124 | 1.941897485 | 0.568330793 | 0.907517089 | 1.08106E-14 | 2.03444E-10 |
| VMP1 | 0.307 | 0.144 | 1.804311242 | 0.524357575 | 0.879445117 | 6.3154E-14 | 1.18849E-09 |
| LAT | 0.528 | 0.229 | 2.842500904 | 1.092999772 | 0.876473445 | 8.97144E-21 | 1.68834E-16 |
| ANKRD10 | 0.376 | 0.132 | 1.791895182 | 0.52347029 | 0.873883413 | 2.51028E-21 | 4.72409E-17 |
| CD84 | 0.261 | 0.08 | 1.38962661 | 0.312696663 | 0.864251631 | 2.05137E-16 | 3.86047E-12 |
| LTB | 0.335 | 0.141 | 2.176941754 | 0.771425894 | 0.842727527 | 1.26214E-14 | 2.37522E-10 |
| CYTH1 | 0.312 | 0.101 | 1.373474795 | 0.348394563 | 0.815758013 | 2.41737E-19 | 4.54925E-15 |

**(B) Downregulated genes (Log2FC ≤ -0.5, pct.1 ≥ 0.25, pct.2** **≤ 0.25)**

| gene | pct.1 | pct.2 | malignant_Mean_UMI_Counts | Other_Mean_UMI_Counts | Log2_Fold_Change | Pvalue | Adjust Pvalue |
| --- | --- | --- | --- | --- | --- | --- | --- |
| GLUL | 0.124 | 0.311 | 0.510964927 | 1.414840982 | -0.676458017 | 6.82995E-10 | 1.28533E-05 |
| ACTN4 | 0.128 | 0.363 | 0.527877387 | 1.53633602 | -0.731217119 | 2.37613E-16 | 4.47164E-12 |
| CTSC | 0.128 | 0.364 | 0.545310258 | 1.62875954 | -0.766485659 | 9.25207E-15 | 1.74115E-10 |
| RGCC | 0.225 | 0.359 | 1.320190141 | 3.04069068 | -0.800358876 | 1.6248E-07 | 0.003057704 |
| RGS1 | 0.179 | 0.363 | 1.281061891 | 3.008834193 | -0.813477157 | 7.89274E-08 | 0.001485335 |
| CST7 | 0.119 | 0.307 | 0.543788727 | 1.966186099 | -0.942133788 | 8.7899E-11 | 1.65417E-06 |
| CXCR4 | 0.161 | 0.332 | 0.71049311 | 2.637681715 | -1.088607025 | 1.33241E-13 | 2.50746E-09 |
| CTSW | 0.041 | 0.258 | 0.206863067 | 1.636200988 | -1.127198373 | 1.99119E-16 | 3.74721E-12 |
| IFNG | 0.06 | 0.299 | 0.419380043 | 2.617223634 | -1.349621869 | 1.96281E-16 | 3.6938E-12 |
| CCL5 | 0.06 | 0.338 | 0.323296296 | 5.051723175 | -2.193209868 | 4.74615E-24 | 8.93177E-20 |

**Abbreviations:** DEGs, Differentially expressed genes.

**Supplementary Table S5. Cell numbers of each donor in each cluster of PBMCs from the SS patient and three HCs for scATAC-seq.**

| **Cell type** | **Cluster** | **Cell numbers of each donor** | | | | **Total of each cluster** |
| --- | --- | --- | --- | --- | --- | --- |
|  |  | **HC-1** | **HC-2** | **HC-3** | **SS** |  |
| malignant CD4^+^ T cells | Cluster 1 | 206 | 225 | 258 | 2033 | 2722 |
| malignant CD4^+^ T cells | Cluster 2 | 5 | 11 | 13 | 2373 | 2402 |
| NK cells | Cluster 3 | 593 | 219 | 689 | 14 | 1515 |
| Benign CD4^+^ T cells | Cluster 4 | 213 | 413 | 365 | 65 | 1056 |
| malignant CD4^+^ T cells | Cluster 5 | 2 | 1 | 1 | 764 | 768 |
| CD8^+^ T cells | Cluster 6 | 45 | 59 | 614 | 23 | 741 |
| NK cells and Myeloid cells | Cluster 7 | 40 | 429 | 48 | 48 | 565 |
| B cells | Cluster 8 | 131 | 140 | 178 | 23 | 472 |
| Benign CD4^+^ T cells and CD8^+^ T cells | Cluster 9 | 1 | 23 | 5 | 429 | 458 |
| undefined | Cluster 10 | 89 | 95 | 58 | 1 | 243 |
| NK cells | Cluster 11 | 18 | 62 | 33 | 3 | 116 |
| Total of each donor | | 1343 | 1677 | 2262 | 5776 | 11058 |

**Supplementary Table S6.**

1. **Demographic and clinical features of MF and SS patients.** Skin lesion and peripheral blood samples of CTCL-3 were used in scRNA-seq, scTCR-seq, and scATAC-seq, while all skin lesions from CTCL patients were used in validation by IHC. Skin lesions from SS patients were used in validation by mIHC. Peripheral blood samples from CTCL-7, CTCL-8 and CTCL-9 were used in validation by qRT-PCR and western blotting.

| Patient no. | Sex | Age | Lesion type | SS or MF category | Disease stage at diagnosis (TNMB) | Sample type |
| --- | --- | --- | --- | --- | --- | --- |
| CTCL-1 | M | 63 | Erythrodemic | SS, IVA2 | T4N3M0B2 | Skin lesion |
| CTCL-2 | M | 31 | Erythrodemic | SS, IVA2 | T4N3M0B2 | Skin lesion |
| CTCL-3 | M | 52 | Erythrodemic | SS, IVA2 | T4N3M0B2 | Skin lesion and peripheral blood |
| CTCL-4 | M | 68 | Erythrodemic | SS, IVA2 | T4N3M0B2 | Skin lesion |
| CTCL-5 | F | 65 | Erythrodemic | SS, IVA2 | T4N3M0B2 | Skin lesion |
| CTCL-6 | M | 68 | Erythrodemic | SS, IVA2 | T4N3M0B2 | Skin lesion |
| CTCL-7 | F | 39 | Tumor | MF, IIB | T3N2M0B0 | Skin lesion and peripheral blood |
| CTCL-8 | F | 64 | Tumor | MF, IIB | T3N1M0B0 | Skin lesion and peripheral blood |
| CTCL-9 | F | 70 | Tumor | MF, IIB | T3N1M0B0 | Skin lesion and peripheral blood |
| CTCL-10 | M | 44 | Tumor | MF, IIB | T3N0M0B0 | Skin lesion |
| CTCL-11 | M | 38 | Tumor | MF, IIB | T3N1M0B0 | Skin lesion |
| CTCL-12 | M | 66 | Plaque | MF, IB | T2N0M0B0 | Skin lesion |
| CTCL-13 | M | 67 | Plaque | MF, IB | T2N0M0B0 | Skin lesion |
| CTCL-14 | M | 37 | Patch | MF, IA | T1N0M0B0 | Skin lesion |
| CTCL-15 | F | 67 | Patch | MF, IB | T2N0M0B0 | Skin lesion |
| CTCL-16 | F | 65 | Patch | MF, IB | T2N0M0B0 | Skin lesion |
| CTCL-17 | F | 37 | Patch | MF, IIA | T2N1M0B0 | Skin lesion |
| CTCL-18 | F | 62 | Patch | MF, IA | T1N0M0B0 | Skin lesion |
| CTCL-19 | M | 29 | Patch | MF, IB | T2N0M0B0 | Skin lesion |

1. **Demographic features of PE patients.** Skin lesions of PE-1 to PE-5 were used in validation by IHC. Peripheral blood samples from PE-6 and PE-7 were used in validation by qRT-PCR and western blotting.

| Patient no. | Sex | Age | Diagnosis | Sample type |
| --- | --- | --- | --- | --- |
| PE-1 | F | 54 | Psoriatic erythroderma | Skin lesion |
| PE-2 | M | 63 | Psoriatic erythroderma | Skin lesion |
| PE-3 | M | 51 | Psoriatic erythroderma | Skin lesion |
| PE-4 | F | 16 | Psoriatic erythroderma | Skin lesion |
| PE-5 | F | 63 | Psoriatic erythroderma | Skin lesion |
| PE-6 | M | 37 | Psoriatic erythroderma | Peripheral blood |
| PE-7 | F | 40 | Psoriatic erythroderma | Peripheral blood |

1. **Demographic features of HCs.** Peripheral blood samples from HC-1 to HC-3 were used for scRNA-seq and scATAC-seq, skin biopsies from HC-4 to HC-6 were used for scRNA-seq, peripheral blood samples from HC-7 to HC-9 were used in validation by flow cytometry, skin biopsies from HC-10 to HC-14 were used in validation by IHC, and peripheral blood samples from HC-15 to HC-16 were used in validation by qRT-PCR and western blotting.

| Subject | Sex | Age | Sample type |
| --- | --- | --- | --- |
| HC-1 | M | 17 | Peripheral blood |
| HC-2 | M | 51 | Peripheral blood |
| HC-3 | F | 52 | Peripheral blood |
| HC-4 | F | 38 | Skin biopsy |
| HC-5 | M | 42 | Skin biopsy |
| HC-6 | M | 55 | Skin biopsy |
| HC-7 | M | 27 | Peripheral blood |
| HC-8 | F | 24 | Peripheral blood |
| HC-9 | F | 34 | Peripheral blood |
| HC-10 | M | 52 | Skin biopsy |
| HC-11 | F | 17 | Skin biopsy |
| HC-12 | F | 27 | Skin biopsy |
| HC-13 | M | 32 | Skin biopsy |
| HC-14 | M | 44 | Skin biopsy |
| HC-15 | F | 29 | Peripheral blood |
| HC-16 | F | 29 | Peripheral blood |

**Supplementary Table S7. Detail information of all antibodies.**

| **Antibody** | **Product ID** | **Distributor** |
| --- | --- | --- |
| APC Mouse Anti-Human CD45 antibody | #555485; | BD Biosciences |
| 7AAD | #559925; | BD Biosciences |
| APC-H7 Mouse Anti-Human CD3 antibody | #560176; | BD Biosciences |
| BV605 Mouse Anti-Human CD4 antibody | #562658; | BD Biosciences |
| PerCP-Cy5.5 Mouse Anti-Human CD45RO antibody | #560607; | BD Biosciences |
| BV421 Mouse Anti-Human CD7 antibody | #562635; | BD Biosciences |
| Human KIR3DL2/CD158k Alexa Fluor® 488-conjugated Antibody | #FAB2878G; | R&D systems |
| Anti- Rabbit- TOX antibody | #ab155768; | Abcam |
| Anti- Rabbit- DNM3 antibody | #ab3458; | Abcam |
| Anti- Rabbit-KLHL42 antibody | #24847-1-AP; | Proteintech |
| Anti- Rabbit- PGM2L1 antibody | #PA5-98545; | Invitrogen |
| Anti- Rabbit- SESN3 antibody | #ab97792; | Abcam |
| Anti- Rabbit- CD4 antibody | #ab133616; | Abcam |
| Anti- Rabbit- GATA3 antibody | #9215; | Cell signaling Technology |
| Anti- Rabbit- Bcl-2 antibody | #ab32124; | Abcam |
| Anti- Rabbit- survivin antibody | #2808S; | Cell signaling Technology |
| Anti- Rabbit-GAPDH antibody | #5852S; | Cell signaling Technology |
| Goat Anti-Rabbit IgG H&L (HRP) antibody | #ZB-2301; | ZSGB-BIO |

**Supplementary Table S8. Details of the immunoreactive scoring system.**

| IRS (SI x PP) | | SI (0-3) | | | |
| --- | --- | --- | --- | --- | --- |
|  |  | Negative | Weak (1) | Moderate (2) | Strong (3) |
| PP (0-4) | Negative (0) | 0 | 0 | 0 | 0 |
|  | ≤25% (1) | 0 | 1 | 2 | 3 |
|  | ≥26%, ≤50% (2) | 0 | 2 | 4 | 6 |
|  | ≥51%, ≤75% (3) | 0 | 3 | 6 | 9 |
|  | ≥76% (4) | 0 | 4 | 8 | 12 |

**Abbreviations:** IRS, immunoreactive score; SI, staining intensity; PP, the percentage of positive cells.

**Supplementary Table S9. The clinical data of 22 MF patients from GSE12902 in GEO.**

| Patient | Gender | Age | Sample | Stage | Follow-up after biopsy(months) |
| --- | --- | --- | --- | --- | --- |
| 1 | M | 67 | GSM323532 | T3N0M0 | 2 |
| 2 | M | 76 | GSM323533 | T3N0M0 | 1 |
| 3 | M | 61 | GSM323534 | T3N0M0 | 45 |
| 4 | M | 39 | GSM323535 | T3N0M0 | 5 |
| 5 | M | 77 | GSM323536 | T3N0M0 | 24 |
| 6 | M | 74 | GSM323537 | T3N0M0 | 24 |
| 7 | M | 80 | GSM323538 | T3N0M0 | 39 |
| 8 | M | 77 | GSM323539 | T3N0M0 | 40 |
| 9 | M | 63 | GSM323540 | T3N0M0 | 41 |
| 10 | M | 69 | GSM323541 | T3N0M0 | 13 |
| 11 | M | 68 | GSM323542 | T3N0M0 | 12 |
| 12 | F | 88 | GSM323543 | T3N0M0 | 3 |
| 13 | M | 59 | GSM323544 | T3N0M0 | 54 |
| 14 | F | 43 | GSM323545 | T3N0M0 | 40 |
| 15 | F | 48 | GSM323546 | T3N0M0 | 4 |
| 16 | M | 75 | GSM323547 | T3N0M0 | 18 |
| 17 | M | 57 | GSM323548 | T3N0M0 | 17 |
| 18 | F | 77 | GSM323549 | T3N0M0 | 41 |
| 19 | M | 54 | GSM323550 | T3N0M0 | 32 |
| 20 | M | 69 | GSM323551 | T3N0M0 | 9 |
| 21 | M | 64 | GSM323552 | T3N3M0 | 11 |
| 22 | M | 67 | GSM323553 | T3N0M0 | 17 |

**Abbreviations**: M, male; F, female.
