# Supplementary figures and images for: Single-cell analyses reveal novel molecular signatures and pathogenesis in cutaneous T cell lymphoma
Source: Cell Death Dis. 2022 Nov 18;13(11):970. doi: 10.1038/s41419-022-05323-5 (PMC9674677; doi:10.1038/s41419-022-05323-5)

Figure 4J

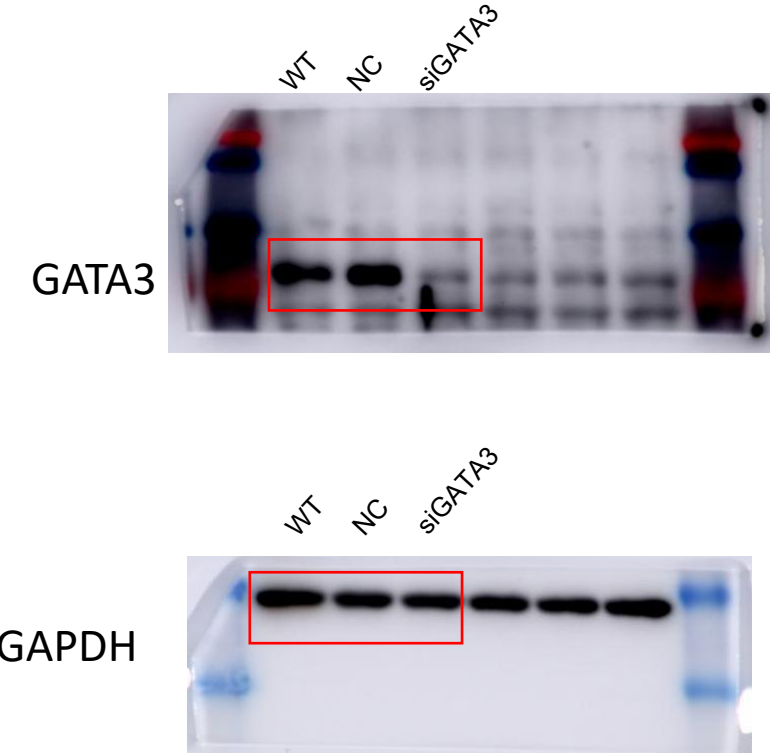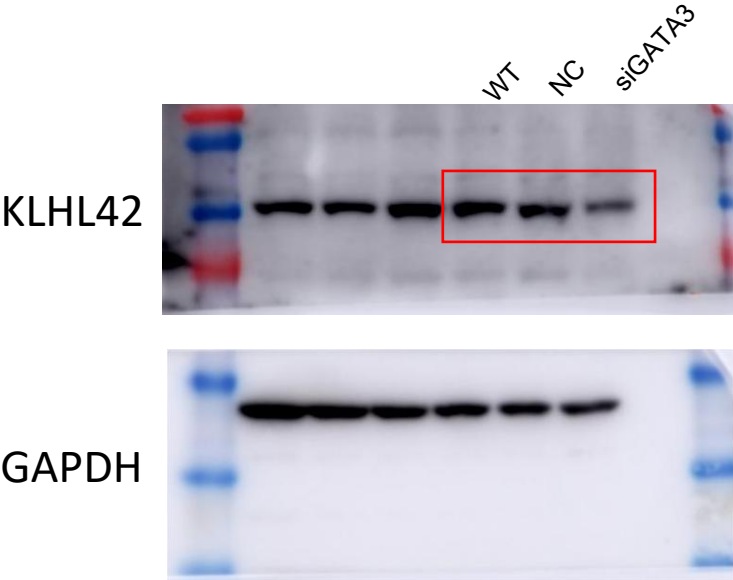

Figure 5B

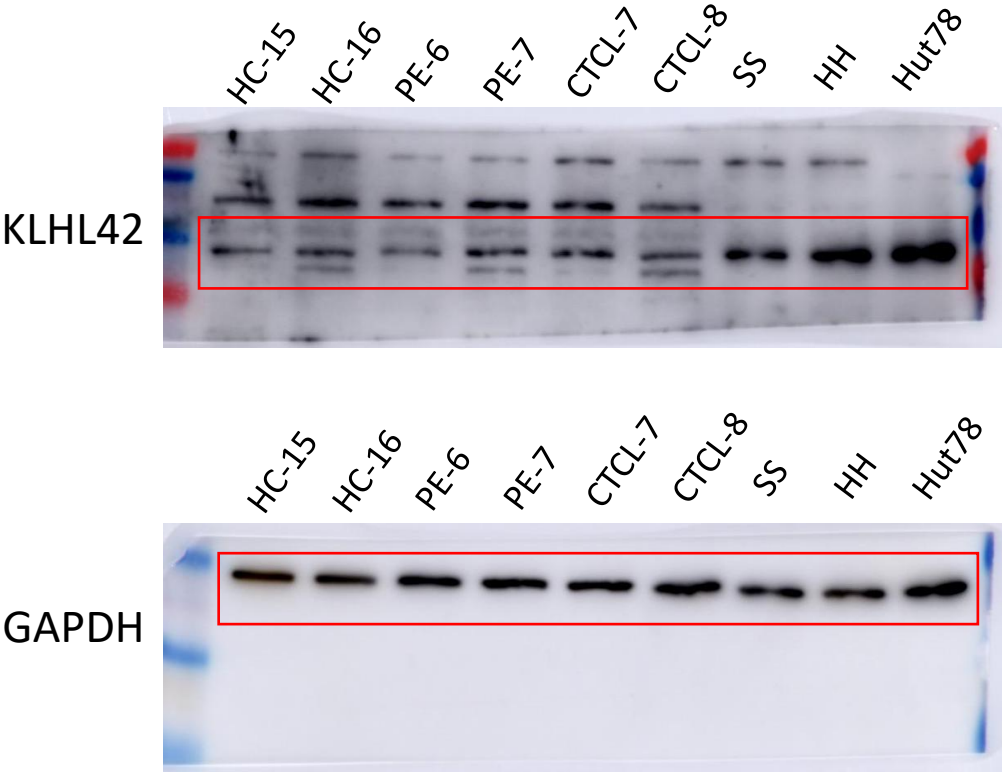

Figure 5D

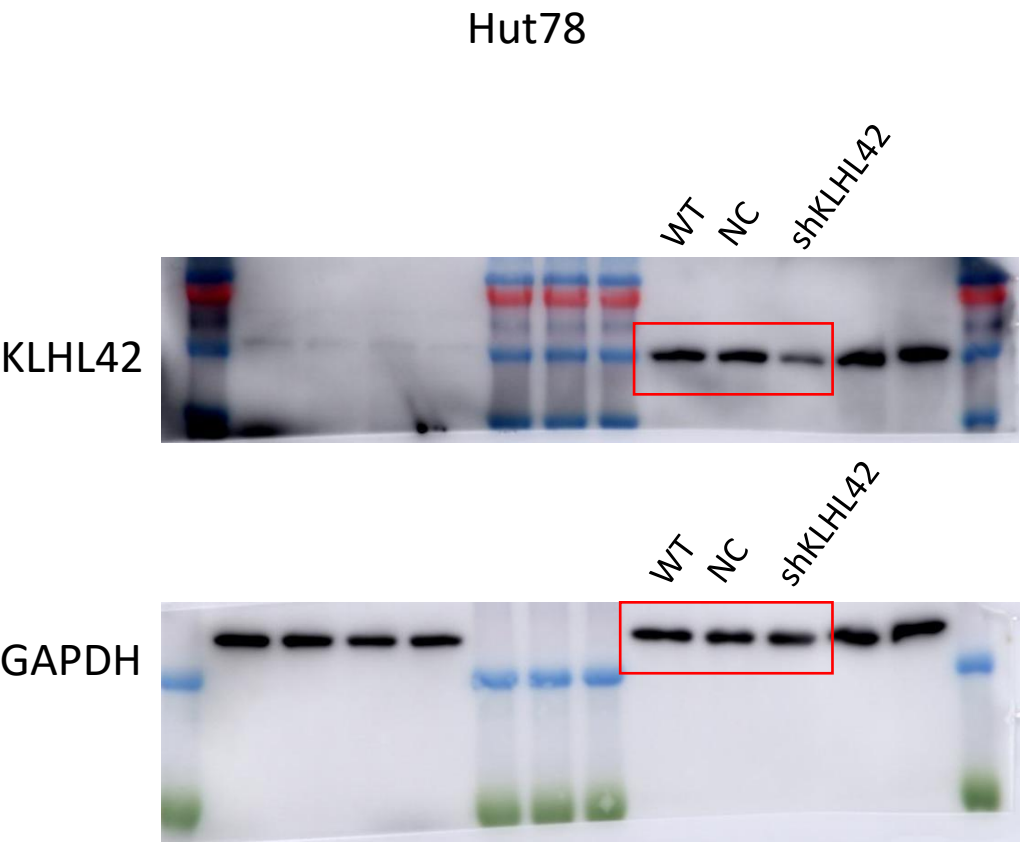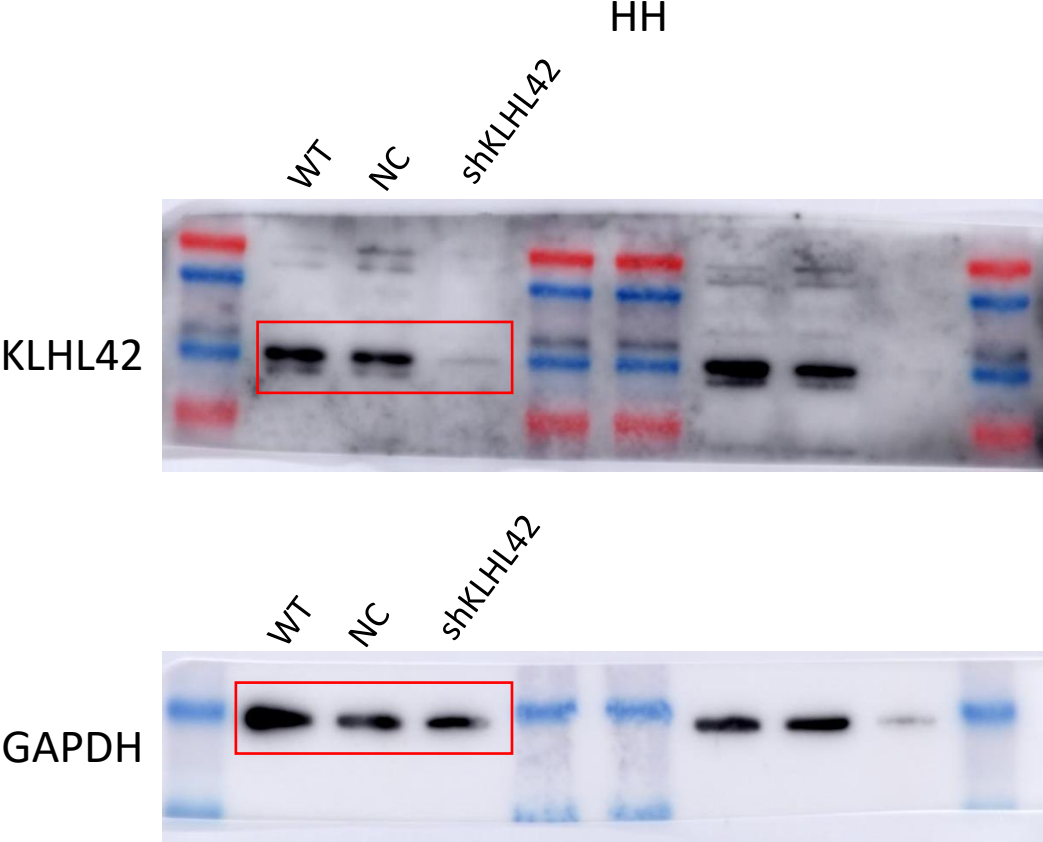

Figure 5F

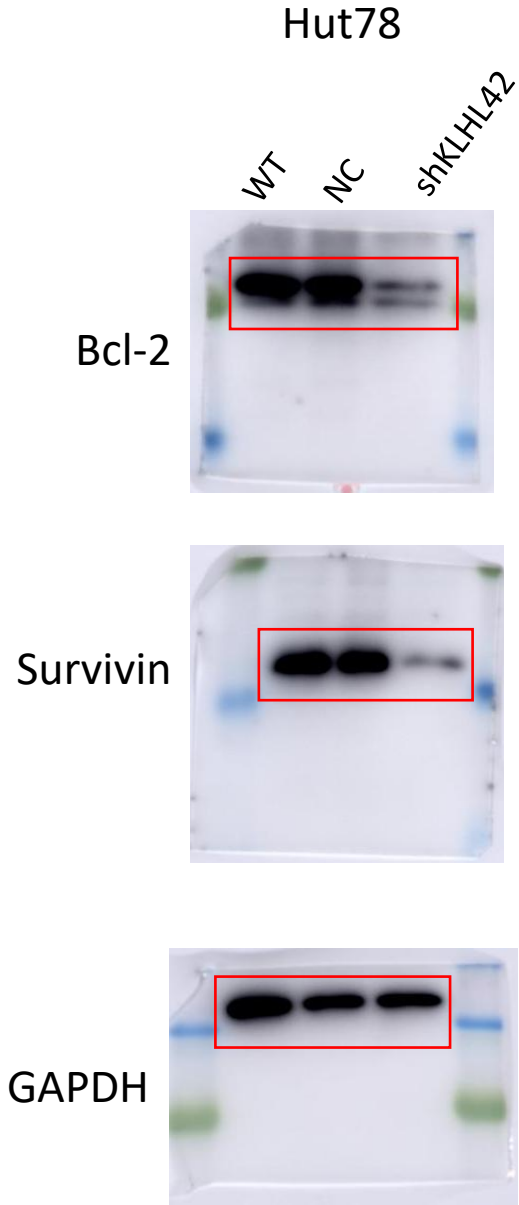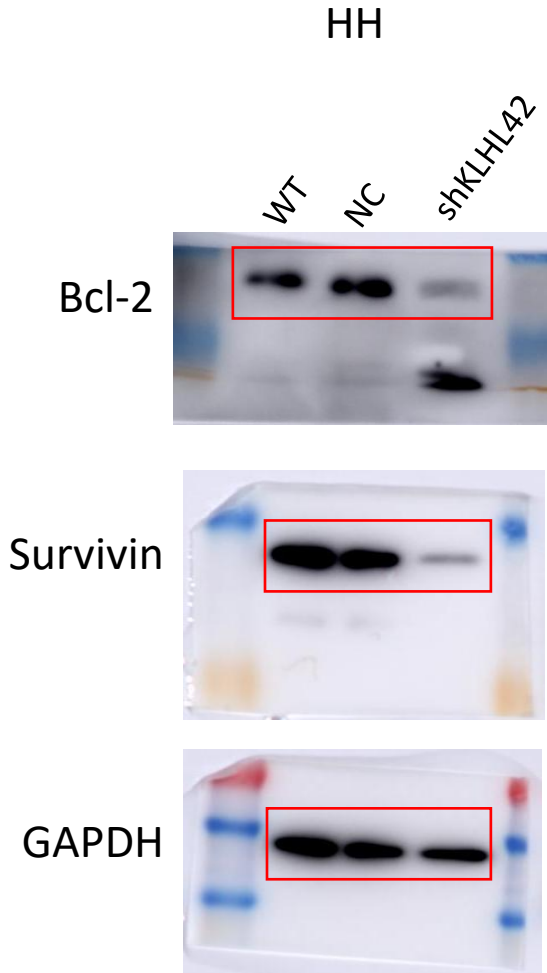

Figure 5l+k

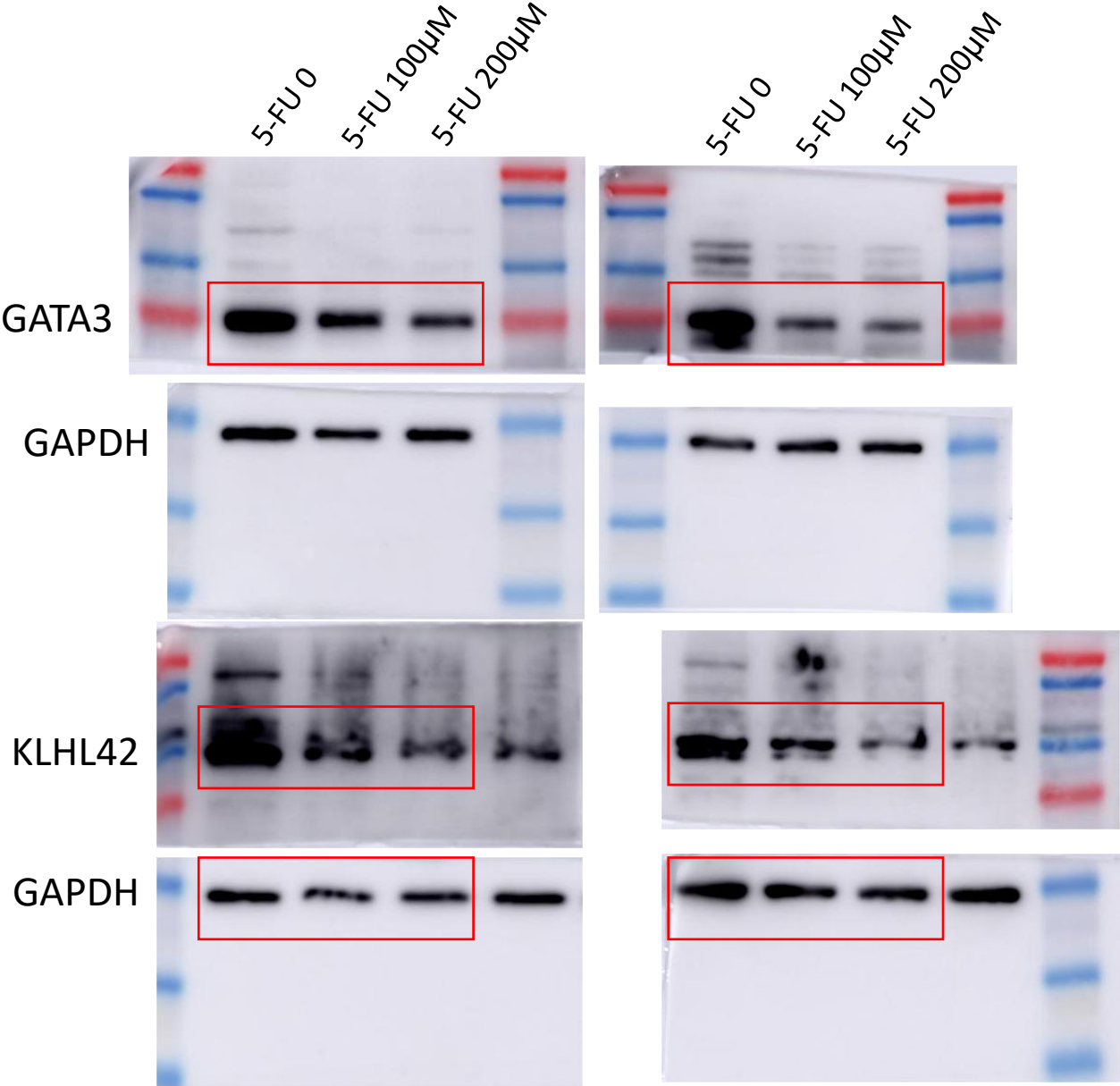

Supplement: Supplementary file 4 — Original Data File [file 41419_2022_5323_MOESM4_ESM.pdf]
